# Understanding Preferences for Preconception Care in Australia: Insights From a Discrete Choice Experiment

**DOI:** 10.1111/hex.70593

**Published:** 2026-02-16

**Authors:** Marion Haas, Jody Church, Edwina Dorney, Deborah J. Street, Kirsten I. Black

**Affiliations:** ^1^ Faculty of Health, Centre for Health Economics Research and Evaluation University of Technology Sydney Sydney Australia; ^2^ Faculty of Medicine and Health, Central Clinical School The University of Sydney Sydney Australia

**Keywords:** consumer preferences, discrete choice experiment, health services research, preconception care, pregnancy, primary care, reproductive health, women's health services

## Abstract

**Background:**

Preconception care (PCC) aims to optimise health before pregnancy by addressing risk factors. This improves conception rates, pregnancy outcomes, and the health of future generations. Despite its benefits, preconception care in Australia is underutilised due to the rate of unplanned pregnancies and inadequate care before pregnancy. Evidence is lacking on consumer preferences for PCC services.

**Methods:**

A discrete choice experiment was designed to investigate preferences of Australian adults for the delivery of PCC services in primary care. An online survey including 12 choice tasks describing alternate PCC service configurations was completed by a sample of the general population. Data were analysed using mixed logit and latent class models to identify key attributes influencing decision‐making and explore preference heterogeneity.

**Results:**

A total of 485 respondents of a generally representative sample of Australian adults completed the online survey. Most respondents (67%) indicated that they had either not heard of PCC or were unsure if they had. Choice modelling results indicated strong preferences for PCC services delivered face‐to‐face, particularly by specialist obstetricians. Lower out‐of‐pocket costs and the inclusion of incentives were also important drivers of preferences.

**Conclusions:**

Increasing awareness of PCC and its importance to the health of parents and babies is essential to improve uptake. Reducing costs and offering incentives may further encourage engagement with PCC services. The results highlight the lack of knowledge concerning the skills of nurse practitioners and pharmacists who are well placed to contribute to PCC delivery in primary care.

## 1 Introduction

Preconception health, the health of a person before pregnancy, impacts conception rates, pregnancy outcomes, childhood health and future generations [1]. Preconception care (PCC) encompasses a range of interventions to optimise health before pregnancy [2] by identification, education and modification of behavioural, biomedical and social risk factors that may affect the health of parents and babies [3]. While beneficial for everyone, certain groups at higher risk (e.g., those with some chronic conditions such as diabetes), groups who are vulnerable (e.g., First Nations communities) or those disadvantaged by lack of access to health care, require targeted interventions [4].

Despite its demonstrated benefits, PCC remains underutilised in Australia. Recent estimates suggest that approximately 30% of pregnancies in Australia are unplanned or unintended [5]. This high prevalence of unplanned pregnancy limits opportunities for individuals to engage in PCC and optimise health before conception. Moreover, evidence on consumer preferences for PCC service delivery is limited, making it difficult to design services that effectively meet community needs and improve uptake.

While many women seek care when pregnant, interventions delivered during pregnancy alone do not maximise health outcomes for women and children [1]. Preconception behaviours such as micronutrient supplementation, strict glycaemic control for diabetes, up‐to‐date vaccinations and minimising environmental exposures or teratogenic medications during this time are key to healthy foetal development [6, 7, 8]. Achieving a healthy weight before conception will improve reproductive outcomes for parents and long‐term outcomes for their children [9, 10].

Despite this evidence, most people do not seek PCC. Many women are initially unaware they are pregnant and do not seek antenatal care before the foetal body systems have developed [11]. Even women with planned pregnancies often lack good preconception behaviours and are unlikely to seek dedicated PCC consultations. Results from a study of women with planned pregnancies attending antenatal care at a large maternity service in Sydney, Australia, showed that 59% had not adopted any preconception behaviours [5]. Although PCC has traditionally focused on women, there is increasing recognition of the important role men play in preconception health, as male health behaviours and medication exposures can influence reproductive and pregnancy outcomes.

Consumers are keen to learn about PCC and expect to be asked about behaviours such as smoking and alcohol [12]. Recent research has explored what people prefer to see in advertisements promoting PCC. While consumers view general practitioners (GPs), midwives and nurses as appropriate providers of PCC, little else is known about the desired features of PCC service delivery [13]. In this study, we used a discrete choice experiment (DCE) to investigate the preferences of the general public, as both potential users and taxpayers, for the delivery of PCC services as part of primary care in Australia. Reflecting a broader, publicly funded healthcare context, the study included respondents of all genders and ages to capture diverse views on PCC service delivery.

## 2 Methods

### 2.1 Discrete Choice Experiments

A DCE is a carefully constructed survey tool in which a hypothetical situation is described. Respondents are then shown sets of possible options (choice tasks) and, for each choice task, are asked to indicate their preferred option. Each option is described by attributes (features or characteristics), and each attribute is presented at one of several possible levels. The choices made are used to understand the preferences of respondents for the characteristics of the attributes and their associated levels [14].

### 2.2 Identification of Attributes and Levels

A focused search of the PCC literature and input from experts in women's reproductive and sexual health were used to develop a list of potential attributes and levels to include in the DCE. The options presented to respondents were PCC consultations described by the type of health care professional (HCP), type of consultation, information provided, incentives and out‐of‐pocket costs (Table 1). We used a generator‐developed (shifted) design [15] to create 8 versions of 12 choice sets from 768 possible PCC consultations, ensuring attribute‐level overlap to prevent simplified decision‐making, with respondents randomised to versions and tasks (see Supporting Information Section S1 for detailed information about the design).

**Table 1:** List of attributes and levels included in the DCE.

Attributes	Levels
Health care professional	General practitioner (GP)
	Nurse practitioner
	Specialist obstetrician
	Pharmacist
Type of consultation	Telehealth consultation
	Face‐to‐face consultation
	Lifestyle questionnaire followed by face‐to‐face consultation
	Lifestyle questionnaire followed by telehealth consultation
What information is provided	A brochure
	A link to a mobile app
	A checklist
	Links to relevant websites
Encouragement to use preconception care	No incentive
	Voucher for recommended pre‐pregnancy supplements
	Sample of pre‐pregnancy vitamins
Out‐of‐pocket costs	$0
	$30
	$50
	$100

### 2.3 Data Collection

**Figure 1:**
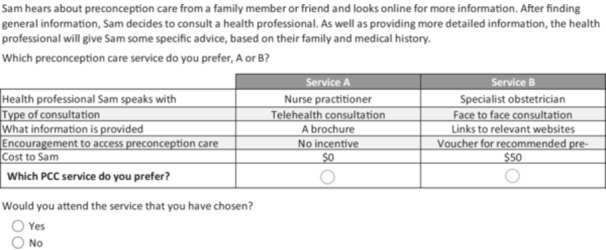
Example of a choice task presented to respondents.

An online survey was developed comprising five parts: (1) questions about age, gender and family dynamics; (2) sociodemographic questions; (3) choice tasks; (4) follow‐up questions; and (5) knowledge of PCC. In the third part of the survey, we asked respondents to imagine a scenario in which an individual was seeking preconception information (Figure 1). Respondents then completed 12 questions comparing two hypothetical PCC services based on the included attributes and levels and chose their preferred option. An additional question after each choice task asked, ‘Would you attend the service that you have chosen?’ to assess the potential uptake of PCC services.

The full survey was first piloted using convenience sampling of researchers with a special interest in DCEs. Once feedback was incorporated and the survey was live, it was paused after 156 responses and again after 262 responses to check for survey flow and logic before the full sample was collected. A version of the full survey is available in Section S9 of the Supporting Information.

### 2.4 Study Sample

**Figure 2:**
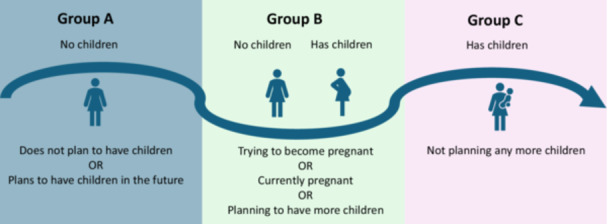
Life stage groups collected in the survey.

The survey was administered by Pureprofile,^1^ a global data and insights organisation which operates a large consumer panel representative of the Australian population. The sample size was determined by collecting at least 20 responses per choice set, as this has demonstrated an acceptable estimate of the standard errors [16]. Data collection occurred in July and August 2023, with respondents compensated by Pureprofile with incentives such as cash, vouchers, gift cards or online payments. Recruitment was based on age and gender quotas, as well as a screening question which asked respondents ‘Do you have children?’, with equal distribution across life stage groups (Figure 2).

Responses were analysed to identify respondents who were not engaged with the survey by flagging speeders (completion time < one‐third of the median completion time), straight‐liners (only chose right‐ or left‐hand side options) or nonsensical responses to open text or feedback questions. Internal validity was assessed by comparing responses to the postal code at the beginning of the survey and the state of residence at the end.

### 2.5 Statistical Analysis

**Figure 3:**
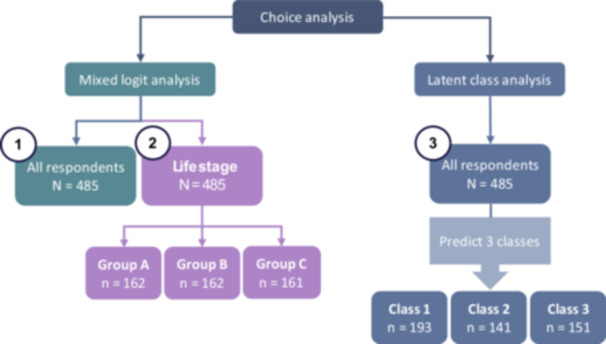
Models fitted in the analysis of choice data.

To account for heterogeneity in preferences, mixed logit models were estimated for all respondents (model 1) and separately by life stage group (model 2). In addition, a latent class analysis (LCA) was conducted (model 3) to identify distinct subgroups (classes) of respondents with similar preferences. The number of classes chosen was based on the lowest Bayesian Information Criterion (BIC) [17]. Respondents were allocated to classes using the highest posterior probability of class membership, and demographic characteristics were explored. All models were estimated using R statistical software (version 4.3.1 [18]), with attribute levels dummy coded using the mlogit package [19] (Figure 3). The DIRECT checklist for DCEs in health guided our study design and reporting [20] (see Supporting Information Section S8, Table S8).

## 3 Results

### 3.1 Characteristics of the Respondents

A total of 485 participants completed the online survey. The study sample was representative of the Australian population in terms of age, gender, Aboriginal and Torres Strait Islander status, income level and state of residence. The sample contained more married respondents, was more highly educated and included more people born in Australia compared with the Australian population (see Supporting Information Section S2, Table S1).

### 3.2 Experience With Preconception Care

Most respondents indicated that they had not heard of PCC (54.6%) or were not sure if they had (12.6%). Respondents who had heard of PCC (*n* = 159, 32.8%) reported hearing about it from family or friends (20.9%), pharmacy (19.1%), e‐mail (18.6%), TV program (16.4%) or internet (15.9%), while only 2.7% had heard of it from a ‘healthcare provider’. Note that multiple responses were possible (see Supporting Information Section S3, Table S2 for answers to PCC questions).

Respondents indicated that a checklist of essential aspects of preconception health (54.4%), a single website that contains all the information, or links to information they needed and can trust (45.8%), and a general brochure on preconception health for distribution to women and men (43.9%) would be useful resources. A mobile app was the least chosen option (30.7%).

To the question regarding which health professional/s could be relied on to deliver high‐quality preconception care, 68.3% responded ‘my GP’, followed by a specialist obstetrician (59.8%), a female GP (42.9%) and a midwife (42.3%). Only 17.3% of respondents indicated that a pharmacist could be relied upon, with a physiotherapist the least chosen option (7.4%).

### 3.3 Analysis of Choice Tasks (Model 1)

Responses to the choice tasks were analysed to identify respondents not engaged with the survey, flagging two speeders (< 1/3 median time of 6.8 min) and two who failed the internal validity test. No respondents ‘straight‐lined’ or provided nonsensical responses. As flagged respondents had similar demographic characteristics to engaged respondents, all respondents were included in the final sample.

**Figure 4:**
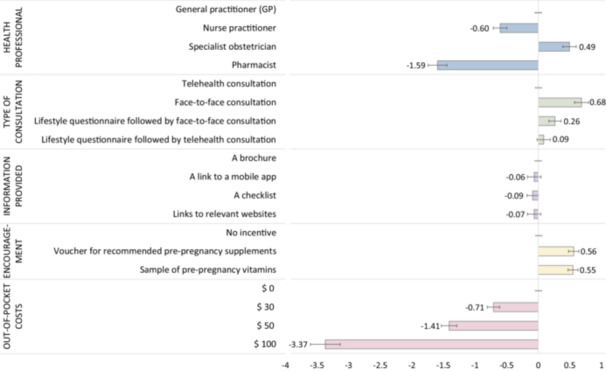
Preferences for the delivery of PCC services. Coefficients to the right of the 0 line indicate preferences above the base level for that attribute; coefficients to the left indicate a preference below the base level.

Analysis showed strong preferences for PCC services delivered by a specialist obstetrician compared with a GP; nurse practitioners and pharmacists were less likely to be preferred. While face‐to‐face consultations were favoured over telehealth, respondents were indifferent to the way information was provided during the consultation. Respondents preferred lower cost services and to receive incentives (vouchers or samples of prenatal vitamins/supplements) as part of PCC, regardless of the type (Figure 4). A table of results can be found in Section S4, Table S3 of the Supporting Information.

### 3.4 Analysis of Choices by Life Stage (Model 2)

Equal numbers of respondents were recruited from the three life stage groups representing their current reproductive/family status and future reproductive plans: A, no children (33.4%); B, planning to have (more) children (33.4%); and C, completed having children (33.2%) (Table 2). As would be expected, 78% of respondents in Group C were over 45 years old (see Table S4).

**Table 2:** Life stage of respondents.

Do you have children?	All participants *N* = 485 *n* (%)
Group A	162 (33.4)
No, but I plan on having children in the future	70 (14.4)
No, I do not plan to have children	92 (19.0)
Group B	162 (33.4)
Yes, and I plan to have more children	128 (26.4)
No, but I am/my partner is currently pregnant	9 (1.9)
No, but I am currently trying to become pregnant	25 (5.2)
Group C	161 (33.2)
Yes, but I do not plan to have any more children	161 (33.2)

**Figure 5:**
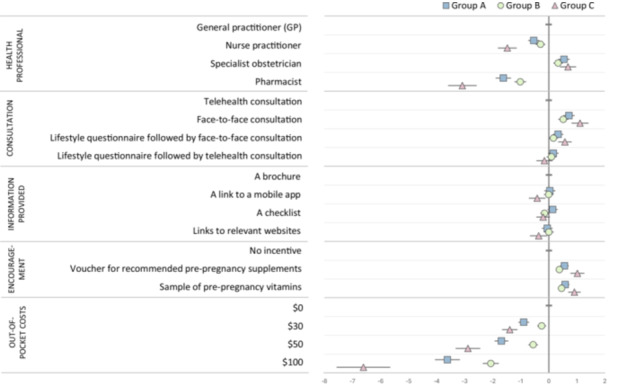
Preferences for PCC by life stage. Coefficients to the right of the 0 line indicate preferences above the base level for that attribute; coefficients to the left indicate a preference below the base level. Overlapping confidence intervals between the groups indicate preferences that are not statistically significantly different.

A MIXL (model 2) estimated preferences for each life stage group independently. Preferences were broadly similar across groups, apart from the HCP delivering PCC and cost. Group C (completed having children) did not favour using nurse practitioners or pharmacists to deliver PCC and disliked higher cost services, which may reflect age‐related differences in healthcare utilisation patterns and greater reliance on traditional, doctor‐led primary care. The cost of services was less important to Group A (no children; see Figure 5). A table of results can be found in Section S5, Table S5 of the Supporting Information.

### 3.5 Latent Class Analysis (Model 3)

The latent class analysis identified three distinct classes based on preferences for PCC services. Class 1 (193 respondents) focused on monetary factors and were averse to higher out‐of‐pocket costs. Class 2 (141 respondents) preferred specialist obstetricians and face‐to‐face consultations and were averse to consultations by nurse practitioners or pharmacists. Class 3 (151 respondents) preferred face‐to‐face consultations and incentives. Demographically, Class 1 had lower education levels and income, Class 2 was older and better educated with higher incomes, and Class 3 was younger and mostly planning to have (more) children (see Supporting Information Section S6, Table S6 and Figure S1).

### 3.6 Feedback From Respondents

Most respondents found the survey ‘very easy’ (12.8%), ‘easy’ (44.3%) or ‘neither easy nor difficult’ (32.8%), with 85.2% indicating that there were ‘about the right number of questions’, compared with ‘too few questions’ (4.1%) or ‘too many questions’ (10.7%). Respondents reported that they focused mainly on out‐of‐pocket costs (59.4%), health professional (54.6%) and type of consultation (50.3%) (see Supporting Information Section S7, Table S7 for a complete table of responses).

## 4 Discussion

This is the first quantitative research in Australia to investigate the preferences of the general public for PCC services. To inform service delivery, we have explored these preferences across the full respondent dataset as well as by life stage; we used a latent class analysis to understand key factors for different cohorts. Although preferences varied across respondents, face‐to‐face consultations with specialist obstetricians or GPs delivered at low cost with incentives were favoured.

Our results show low levels of awareness and adoption rates for PCC; 67% of respondents had either not heard of or were unsure if they had heard of PCC. This indicates a need for increased efforts to raise awareness about PCC and its importance, consistent with findings reported in Australia and internationally [12, 21, 22, 23, 24]. Only 2% of respondents who knew about PCC had heard about it from an HCP, which may reflect the fact that only 53% of GPs are aware of the RACGP and/or the Royal Australian and New Zealand College of Obstetricians and Gynaecologists (RANZCOG) pre‐pregnancy counselling clinical guidelines [25].

Raising awareness of PCC and its importance to the health of parents and babies is vital to encourage uptake of PCC services. Both local and international data [26, 27, 28] show that among women who plan a pregnancy, less than 50% see a health professional for PCC, with some rates as low as 17% [29]. Women who are least likely to adopt preconception behaviours or seek PCC tend to be younger, have more children (higher parity) and do not have private health insurance [26]. Research has demonstrated that women who receive preconception health information are more likely to make healthier lifestyle changes, such as quitting alcohol and smoking, improving their nutrition and taking folic acid supplements [12]. According to the National Health Survey, about 19% of females aged 18–44 exceed the recommended guidelines for alcohol consumption [30].

Respondents thought that different HCPs (their GP, a specialist gynaecologist/obstetrician, a female GP or a midwife) could deliver high‐quality PCC, indicating trust in HCPs once awareness is established. Respondents were open to receiving general comprehensive PCC information in a variety of forms (e.g., checklist, single website, multiple links, brochure). These results show that PCC can be delivered flexibly, allowing for local needs to be considered and for multiple formats to be used to reinforce PCC messages.

Despite indicating that midwives (42.3%) and RNs (38.4%) could be relied on to deliver high‐quality PCC, in the choice tasks, respondents were averse to provision by practitioners other than specialist obstetricians and GPs. Information and education regarding the expertise and skills of other HCPs are needed, as they could be valuable sources of general and specific information on PCC. In the same way that practice nurses, NPs and pharmacists have become trusted providers of vaccinations, they undoubtedly have the necessary professional proficiency to deliver PCC. Traditionally in Australia, there has been more focus on nurses delivering PCC rather than midwives in the primary care setting; therefore, midwives were not included in the choice tasks. As there are increased efforts to involve midwives in both primary care and the delivery of PCC [31], respondents were asked about the role of midwives in PCC in follow‐up questions. The results of these stated preferences support the involvement of midwives in PCC.

Cost was an important issue for respondents, particularly those with lower incomes. To address this in the Australian context, incentivising HCPs working in primary care to provide PCC through Medicare (Australia's publicly funded universal health insurance scheme) could improve affordability, for example, by supporting services in family planning clinics, general practices and pharmacies. An initial investment in PCC is likely to be offset by improved maternal and child health outcomes, thus reducing downstream healthcare costs. The latent class analysis revealed that higher‐income respondents were less affected by cost and preferred services with a specialist obstetrician; the cost of PCC services is a significant barrier for lower‐income groups. Incentives, such as vouchers or samples of prenatal vitamins/supplements, may encourage women to attend PCC services.

Despite policy initiatives in Australia designed to enhance the availability of PCC services, only a few isolated hospital‐based PCC services exist that allow GP referrals, and currently, no comprehensive PCC programs are available for individuals or couples planning to have children. GPs can play a pivotal role in enhancing access to preconception health assessments and counselling for both women and men [32]. Recent regulatory guidance in Australia has highlighted potential risks associated with paternal exposure to medications such as sodium valproate [33], reinforcing the importance of engaging men in PCC and underscoring a critical gap in current service delivery.

Our study underscores the importance of targeted interventions for high‐risk and vulnerable groups, such as those with chronic conditions or limited access to healthcare. Tailoring PCC services to meet the needs of these groups can improve health outcomes.

The research has some limitations. As DCEs include a limited number of attributes, some aspects of PCC delivery, important to some respondents, may be missing, although feedback did not highlight any omissions. The respondent panel was generally representative of the Australian population, but overall were more likely to be highly educated, married and born in Australia. Approximately half of the respondents were aged over 45 years, and most had completed having children. Although this group may not reflect the preferences of individuals currently planning a pregnancy, their views remain relevant for PCC policy design, as they may represent partners, previous service users or broader community preferences that influence policy acceptability. However, preferences among younger individuals of reproductive age may differ and should be considered when interpreting the findings. Additionally, the lack of data on nonrespondents prevented assessment of response bias, further limiting the generalisability of the results. Strengths of the research include the careful design of the DCE with input from previous research and experts in the field. Most respondents found the survey easy to complete and were representative of the age and gender of the Australian population. Similar responses to the choice tasks and the follow‐up questions about which attributes were focused on indicate both an understanding of and engagement with the survey. Over 20% of respondents provided positive feedback comments, indicating that they found the survey enjoyable and informative.

This study provides valuable insights into preferences for PCC services in Australia. Despite low awareness of PCC, respondents preferred services delivered by specialist obstetricians and GPs, with a strong emphasis on face‐to‐face consultations and lower cost services. The findings highlight the need for increased awareness and accessibility, particularly for lower‐income and high‐risk groups. Tailoring PCC services to meet diverse needs and leveraging various healthcare providers can enhance uptake and improve health outcomes for parents and children. Future research should continue to explore effective strategies for promoting and delivering PCC.

## Author Contributions

**Marion Haas:** conceptualisation, methodology, validation, supervision, writing – original draft, writing – review and editing. **Jody Church:** conceptualisation, methodology, data curation, formal analysis, validation, visualisation, writing – original draft, writing – review and editing. **Edwina Dorney:** conceptualisation, data curation, visualisation, writing – original draft, writing – review and editing. **Deborah J. Street:** conceptualisation, methodology, data curation, formal analysis, validation, writing – original draft, writing – review and editing. **Kirsten I. Black:** conceptualisation, methodology, writing – original draft, writing – review and editing.

## Ethics Statement

Ethics approval was granted by the University of Technology Sydney Human Research Ethics Committee (HREC Reference No. ETH18‐2507).

## Conflicts of Interest

The authors declare no conflicts of interest.

## Patient or Public Contribution

Members of the public participated in our study by completing a discrete choice experiment and a survey administered by Pureprofile, an online panel provider (pureprofile.com). Respondents were not involved in the design, conduct, analysis, or interpretation of the results; however, their responses and opinions asked in the survey informed our study findings.

## Supporting information

3_PCC_DCE_Supporting_Information_FINAL_v16.

## Data Availability

The data that support the findings of this study are available from the corresponding author upon reasonable request.

## Endnotes

1 Pureprofile Australia Pty Limited: www.pureprofile.com
