# Supplementary material for: Understanding Preferences for Preconception Care in Australia: Insights From a Discrete Choice Experiment
Source: Health Expect. 2026 Feb 16;29(1):e70593. doi: 10.1111/hex.70593 (PMC12909604; doi:10.1111/hex.70593)
Supplement: Supplementary file 1 — 3_PCC_DCE_Supporting_Information_FINAL_v16. [file HEX-29-e70593-s001.docx]

**SUPPORTING INFORMATION**

**TITLE**: Understanding Australian preferences for preconception care: Insights from a discrete choice experiment

**AUTHORS**: M Haas, J Church, E Dorney, DJ Street, KI Black

Table of Contents

[1 Details about the designed experiment 2](#_Toc218948326)

[2 Demographics of respondents 3](#_Toc218948327)

[3 Experience with preconception care questions 4](#_Toc218948328)

[4 Mixed logit model: all respondents (model 1) 5](#_Toc218948329)

[5 Mixed logit model: by life stage group (model 2) 6](#_Toc218948330)

[6 Latent class analysis (model 3) 7](#_Toc218948331)

[7 Feedback questions 9](#_Toc218948332)

[8 DIRECT checklist 9](#_Toc218948333)

[9 Example of a version of the full survey 11](#_Toc218948334)

Supplementary Tables

[Table S1. Respondent Demographics 3](#_Toc218948426)

[Table S2. Preconception Care 4](#_Toc218948427)

[Table S3. Mixed logit model: all respondents 5](#_Toc218948428)

[Table S4. Ages of respondents by life stage groups 5](#_Toc218948429)

[Table S5. Mixed logit models: by life stage group 6](#_Toc218948430)

[Table S6. Latent class analysis 7](#_Toc218948431)

[Table S7. Feedback Questions 9](#_Toc218948432)

[Table S8. Checklist for reporting discrete choice experiments in health 9](#_Toc218948433)

Supplementary FIGURES

[Figure S1. Preferences for PCC by class………………………………………………………………. 8](#_Toc194049249)

# Details about the designed experiment

The number of PCC consultations that could be described, given all the possible combinations of attributes and levels, would be 768 (four attributes with four levels and one attribute with 3 levels). There were no implausible combinations of attributes and levels that needed to be accounted for. We used a generator-developed (shifted) design that included choice sets with attribute-level overlap on exactly one attribute and some choice sets in which none of the attributes overlapped. This approach prevented respondents from using a simplified decision-making strategy by focusing on only one attribute. The initial orthogonal array was the 4^5 in 16 runs from Kuhfeld’s site^[[1]](#footnote-2)^ and one of the 4-level attributes was collapsed to 3 levels to account for the three levels of the encouragement attribute. Although we know that it is statistically most efficient to have non-zero entries in each position of the generators, we would also prefer to stop people from developing a choice heuristic that only considers one of the attributes and so in total 6 generators were used, with one generator containing all non-zero entries and with each attribute having a 0 entry in exactly one of the other generators. The resulting design had a D-efficiency of 83.04% under the null prior and a D-error of 0.1142. These 96 choice sets were subdivided into 8 versions of 12 choice sets each, with each version containing two choice sets arising from each of the generators with no overlap and two choice sets with overlap on each of the five attributes. Respondents were randomised to one of these versions. The order of the choice tasks within each version was randomised, as was the order of service A and service B.

# Demographics of respondents

Table S1. Respondent Demographics

|  | All participants (N =485)  n (%) | Australian Population (%) |
| --- | --- | --- |
| Age Groups^a^ |  |  |
| 18 - 24 years | 60 (12.4) | 11.0 |
| 25 - 29 years | 41 (8.5) | 9.0 |
| 30 - 34 years | 52 (10.7) | 9.4 |
| 35- 39 years | 49 (10.1) | 9.3 |
| 40 - 44 years | 49 (10.1) | 8.4 |
| 45+ years | 234 (48.3) | 52.8 |
| Gender^a^* |  |  |
| Male | 249 (51.3) | 49.1 |
| Female | 235 (48.5) | 50.9 |
| Other | 1 (0.2) | - |
| Country of birth^b^ |  |  |
| Australia | 388 (80.0) | 70.9 |
| Overseas | 97 (20.0) | 29.1 |
| Aboriginal or Torres Strait Islander status^c^ |  |  |
| No | 458 (94.4) | 96.8 |
| Yes - Aboriginal, Torres Strait Islander, both | 27 (5.6) | 3.2 |
| Place of residence^d^ |  |  |
| Metropolitan | 353 (72.8) | 73.0 |
| Rural | 132 (27.2) | 27.0 |
| Education^e^ |  |  |
| Year 11 and below | 43 (8.9) | 21.4 |
| Year 12 | 81 (16.7) | 17.5 |
| Certificate (any level) | 79 (16.3) | 16.8 |
| Diploma / advanced | 61 (12.6) | 10.3 |
| Bachelor / honours | 150 (30.9) | 20.0 |
| Post graduate degree | 71 (14.6) | 12.1 |
| Household income^f^ |  |  |
| $0 - $77,999 per year ($0 - $1499) | 167 (34.4) | 42.3 |
| $78,000 - $155,999 per year ($1500 - $2999) | 176 (36.3) | 32.0 |
| $156,000 - $259,999 per year ($3000 - $4999) | 87 (18.0) | 17.9 |
| $260,000 or more per year ($5000 +) | 32 (6.6) | 7.7 |
| Don’t know / Prefer not to say | 23 (4.7) | - |
| Marital status^g^ |  |  |
| Never married | 122 (25.2) | 36.5 |
| Married or de facto | 310 (63.9) | 46.5 |
| Separated/Divorced/Widowed | 53 (10.9) | 17.1 |

*Gender data pertains to adults aged ≥18 years.
^a^Australian Bureau of Statistics. "National, state and territory population." ABS, March 2024, <https://www.abs.gov.au/statistics/people/population/national-state-and-territory-population/latest-release>.

^b^Australian Bureau of Statistics. "Australia's Population by Country of Birth." *ABS*, June 2023, <https://www.abs.gov.au/statistics/people/population/australias-population-country-birth/latest-release>.

^c^Australian Bureau of Statistics. "Aboriginal and Torres Strait Islander people: Census." *ABS*, 2021, <https://www.abs.gov.au/statistics/people/aboriginal-and-torres-strait-islander-peoples/aboriginal-and-torres-strait-islander-people-census/latest-release>.

^d^Australian Institute of Health and Welfare. “Profile of Australia's population.” AIHW, April 2024, <https://www.aihw.gov.au/reports/australias-health/profile-of-australias-population>

^e^Australian Bureau of Statistics. "Education and Work, Australia." *ABS*, May 2024, <https://www.abs.gov.au/statistics/people/education/education-and-work-australia/latest-release>.

^f^Australian Bureau of Statistics. "Household Income and Wealth, Australia." *ABS*, 2019-20, <https://www.abs.gov.au/statistics/economy/finance/household-income-and-wealth-australia/latest-release>.

^g^Australian Bureau of Statistics. "Household and families: Census." *ABS*, 2021, <https://www.abs.gov.au/statistics/people/people-and-communities/household-and-families-census/2021>.

# Experience with preconception care questions

Table S2. Preconception Care

|  | All Participants (N = 485)  n(%) |
| --- | --- |
| Have you heard of pre-conception care? |  |
| No | 265 (54.6) |
| Yes | 159 (32.8) |
| Not sure | 61 (12.6) |
| If you have seen or heard anything about preconception care, where was it? |  |
| Family or friend | 46 (20.9) |
| Nothing recently | 46 (20.9) |
| Pharmacy | 42 (19.1) |
| E-mail | 41 (18.6) |
| TV program | 36 (16.4) |
| Internet | 35 (15.9) |
| Hospital | 30 (13.6) |
| Radio | 22 (10.0) |
| Newspapers | 21 (9.6) |
| A mobile app | 21 (9.6) |
| Social media | 16 (7.3) |
| Not sure | 18 (8.2) |
| Blog | 10 (4.6) |
| Healthcare provider | 6 (2.7) |
| Have you or your partner received preconception care (e.g. prior to a previous pregnancy)? |  |
| No | 125 (56.8) |
| Yes | 68 (30.9) |
| Not sure | 27 (12.3) |
| Has a health care professional (doctor, nurse etc.) ever told you/ your partner that you should get special medical care and advice before becoming pregnant? |  |
| No | 119 (54.1) |
| Yes | 78 (35.5) |
| Not sure | 23 (10.5) |
| If receiving preconception health information, what kinds of resources would you find useful? |  |
| A checklist of essential aspects of preconception health | 264 (54.4) |
| A single website that contains all the information, or links to information, you need and can trust | 222 (45.8) |
| General brochure on preconception health targeted for distribution to women and men | 213 (43.9) |
| A list of websites with information you can trust | 177 (36.5) |
| General brochure on preconception health targeted for distribution to women | 165 (34.0) |
| A mobile app | 149 (30.7) |
| Not sure | 55 (11.3) |
| Other | 2 (0.4) |
| Which of the following people do you think could be relied on to deliver high quality preconception care? |  |
| My GP | 331 (68.3) |
| A specialised obstetrician | 290 (59.8) |
| A female GP | 208 (42.9) |
| A midwife | 205 (42.3) |
| A registered nurse | 186 (38.4) |
| Family planning clinics | 161 (33.2) |
| A pharmacist | 84 (17.3) |
| Any medical specialist | 38 (7.8) |
| A physiotherapist | 36 (7.4) |
| Other | 4 (0.8) |

# Mixed logit model: all respondents (model 1)

Table S3. Mixed logit model: all respondents

|  | **M1: Mixed logit model** | |
| --- | --- | --- |
|  | Coefficient (SE) | *Std. Dev*  *Mean (SE)* |
| Health professional |  |  |
| GP | - *reference level* |  |
| Nurse practitioner | -0.60 (0.11)*** | *1.19 (0.18)**** |
| Specialist obstetrician | 0.49 (0.11)*** | *1.65 (0.19)**** |
| Pharmacist | -1.59 (0.15)*** | *1.84 (0.19)**** |
| **Consultation** |  |  |
| Telehealth consultation | - *reference level* |  |
| Face-to-face consultation | 0.68 (0.11)*** | *0.99 (0.17)**** |
| Lifestyle questionnaire / face-to-face | 0.26 (0.10)** | *1.25 (0.16)**** |
| Lifestyle questionnaire /telehealth | 0.09 (0.10) | *0.47 (0.24)* |
| Information |  |  |
| A brochure | *- reference level* |  |
| A link to a mobile app | -0.06 (0.11) | *0.64 (0.22)*** |
| A checklist | -0.09 (0.09) | *0.58 (0.21)*** |
| Links to relevant websites | -0.07 (0.11) | *0.66 (0.21)*** |
| **Incentives** |  |  |
| No incentive | - *reference level* |  |
| Voucher for supplements | 0.56 (0.08)*** | *0.93 (0.15)**** |
| Sample of pre-pregnancy vitamins | 0.55 (0.08)*** | *0.48 (0.20)** |
| **Out of pocket costs** |  |  |
| $0 | - *reference level* | *-* |
| $30 | -0.71 (0.10)*** | *0.23 (0.36)* |
| $50 | -1.41 (0.12)*** | *1.22 (0.18)**** |
| $100 | -3.37 (0.23)*** | *2.45 (0.23)**** |
| Log-likelihood |  | -3,111 |
| AIC |  | 6,278 |

GP: General practitioner, AIC: Akaike Information Criterion.
Statistical significance: * p ≤ 0.05, ** p ≤ 0.01, *** p ≤ 0.001

Table S4. Ages of respondents by life stage groups

| Age band (years) | Group A: No children  n (%) | Group B: Planning children n (%) | Group C: Completed having children n (%) |
| --- | --- | --- | --- |
| 18–24 | 24 (15%) | 35 (22%) | 1 (1%) |
| 25–29 | 18 (11%) | 21 (13%) | 2 (1%) |
| 30–34 | 13 (8%) | 33 (20%) | 6 (4%) |
| 35–39 | 20 (12%) | 19 (12%) | 10 (6%) |
| 40–44 | 11 (7%) | 22 (14%) | 16 (10%) |
| ≥45 | 76 (47%) | 32 (20%) | 126 (78%) |
| Total | **162 (100%)** | **162 (100%)** | **161 (100%)** |

# Mixed logit model: by life stage group (model 2)

Table S5. Mixed logit models: by life stage group

|  | **M2: Mixed logit model by life stage** | | | | | |
| --- | --- | --- | --- | --- | --- | --- |
|  | Group A  (No children) | | Group B  (Planning children) | | Group C  (Done having children) | |
|  | Coefficient (SE) | *Std. deviation (SE)* | Coefficient (SE) | *Std. deviation (SE)* | Coefficient (SE) | *Std. deviation (SE)* |
| Health professional |  |  |  |  |  |  |
| GP | - *reference level* |  |  |  |  |  |
| Nurse practitioner | -0.54 (0.19)** | *1.12 (0.32)**** | -0.30 (0.16) | *1.30 (0.27)**** | -1.48 (0.33)*** | *1.31 (0.47)*** |
| Specialist obstetrician | 0.54 (0.19)** | *1.55 (0.32)**** | 0.33 (0.16)* | *1.30 (0.28)**** | 0.68 (0.28)* | *2.97 (0.56)**** |
| Pharmacist | -1.62 (0.26)*** | *2.16 (0.36)**** | -1.02 (0.20)*** | *1.32 (0.28)**** | -3.08 (0.50)*** | *2.56 (0.49)**** |
| **Consultation** |  |  |  |  |  |  |
| Telehealth consultation | - *reference level* |  |  |  |  |  |
| Face-to-face consultation | 0.72 (0.19)*** | *1.03 (0.29)**** | 0.51 (0.17)** | *0.88 (0.27)*** | 1.11 (0.29)*** | *0.42 (0.68)* |
| Lifestyle questionnaire / face-to-face | 0.33 (0.17) | *1.33 (0.30)**** | 0.16 (0.15) | *0.97 (0.26)**** | 0.57 (0.23)* | *2.11 (0.40)**** |
| Lifestyle questionnaire /telehealth | 0.16 (0.18) | *0.69 (0.33)** | 0.10 (0.16) | *0.45 (0.40)* | -0.16 (0.28) | *1.75 (0.41)**** |
| Information |  |  |  |  |  |  |
| A brochure | - *reference level* |  |  |  |  |  |
| A link to a mobile app | 0.04 (0.19) | *0.77 (0.34)** | 0.005 (0.16) | *0.28 (0.59)* | -0.41 (0.29) | *1.29 (0.39)**** |
| A checklist | 0.14 (0.17) | *0.04 (1.05)* | -0.15 (0.14) | *0.59 (0.31)* | -0.20 (0.23) | *0.81 (0.42)* |
| Links to relevant websites | -0.06 (0.18) | *0.29 (0.67)* | 0.003 (0.16) | *0.78 (0.31)** | -0.36 (0.30) | *1.25 (0.40)*** |
| **Incentives** |  |  |  |  |  |  |
| No incentive | - *reference level* |  |  |  |  |  |
| Voucher for supplements | 0.56 (0.14)*** | *0.62 (0.32)** | 0.38 (0.12)** | *0.95 (0.23)**** | 1.02 (0.24)*** | *1.77 (0.39)**** |
| Sample of pre-pregnancy vitamins | 0.58 (0.14)*** | *0.74 (0.29)** | 0.45 (0.12)*** | *0.26 (0.53)* | 0.91 (0.21)*** | *0.87 (0.39)** |
| **Out of pocket costs** |  |  |  |  |  |  |
| $0 | - *reference level* |  | - |  |  |  |
| $30 | -0.89 (0.18)*** | *0.19 (0.78)* | -0.25 (0.15) | *0.66 (0.34)* | -1.39 (0.26)*** | *0.22 (0.38)* |
| $50 | -1.69 (0.23)*** | *0.72 (0.35)** | -0.56 (0.16)*** | *1.10 (0.29)**** | -2.88 (0.43)*** | *2.14 (0.43)**** |
| $100 | -3.62 (0.43)*** | *2.48 (0.43)**** | -2.07 (0.28)*** | *1.83 (0.32)**** | -6.61 (0.94)*** | *3.59 (0.64)**** |
| Log-likelihood | -1,011.10 | | -1,156.10 | | -885.07 | |
| AIC | 2,078.25 | | 2,368.25 | | 1,826.14 | |

GP: General practitioner, AIC: Akaike Information Criterion.
Statistical significance: * p ≤ 0.05, ** p ≤ 0.01, *** p ≤ 0.001

# Latent class analysis (model 3)

Table S6. Latent class analysis

|  | **M3: Latent Class Analysis** | | |
| --- | --- | --- | --- |
|  | Class 1  Coefficient (SE) | Class 2  Coefficient (SE) | Class 3  Coefficient (SE) |
| Health professional |  |  |  |
| GP | - *reference level* |  |  |
| Nurse practitioner | -0.26 (0.16) | -1.53 (0.19)*** | 0.07 (0.11) |
| Specialist obstetrician | 0.05 (0.16) | 1.47 (0.24)*** | 0.03 (0.11) |
| Pharmacist | -0.88 (0.18)*** | -3.12 (0.30)*** | -0.09 (0.12) |
| **Consultation** |  |  |  |
| Telehealth consultation | - *reference level* |  |  |
| Face-to-face consultation | 0.12 (0.14) | 1.08 (0.19)*** | 0.44 (0.12)*** |
| Lifestyle questionnaire / face-to-face | -0.06 (0.14) | 0.94 (0.17)*** | 0.12 (0.11) |
| Lifestyle questionnaire /telehealth | -0.01 (0.14) | 0.32 (0.21) | 0.14 (0.11) |
| Information |  |  |  |
| A brochure | - *reference level* |  |  |
| A link to a mobile app | 0.01 (0.18) | -0.39 (0.22) | -0.01 (0.10) |
| A checklist | -0.04 (0.13) | 0.22 (0.17) | 0.004 (0.10) |
| Links to relevant websites | 0.04 (0.18) | -0.08 (0.23) | 0.01 (0.11) |
| **Incentives** |  |  |  |
| No incentive | - *reference level* |  |  |
| Voucher for supplements | 0.87 (0.12)*** | 0.11 (0.16) | 0.21 (0.08)* |
| Sample of pre-pregnancy vitamins | 0.64 (0.12)*** | 0.35 (0.14)* | 0.17 (0.08)* |
| **Out of pocket costs** |  |  |  |
| $0 | - *reference level* | - |  |
| $30 | -1.64 (0.20)*** | -0.21 (0.19) | 0.12 (0.11) |
| $50 | -2.62 (0.26)*** | -0.60 (0.20)** | 0.11 (0.11) |
| $100 | -5.03 (0.39)*** | -1.90 (0.28)*** | -0.22 (0.15) |
| Class 2 | -0.38 (0.04)*** | | |
| Class 3 | -0.20 (0.05)*** | | |
| Class share | 39.8% | 29.1% | 31.1% |
| Log-likelihood |  |  | -3,026.90 |
| AIC |  |  | 6,141.71 |

GP: General practitioner, AIC: Akaike Information Criterion.
Statistical significance: * p ≤ 0.05, ** p ≤ 0.01, *** p ≤ 0.001; The median posterior probability was 97.4%, indicating that the model was able to distinguish different preference patterns within the choice data.

**Figure S1.** **Preferences for PCC by class.** Coefficients to the right of the 0 line indicate preferences above the base level for that attribute; coefficients to the left indicate a preference below the base level. Overlapping confidence intervals between the groups indicate preferences that are not statistically significantly different.

# Feedback questions

Table S7. Feedback Questions

|  | All participants  N = 485  n (%) |
| --- | --- |
| How easy or difficult did you find answering the 12 choice questions? |  |
| Very easy | 62 (12.8) |
| Easy | 215 (44.3) |
| Neither easy nor difficult | 159 (32.8) |
| Difficult | 45 (9.3) |
| Very difficult | 4 (0.8) |
| In terms of the number of choice questions, what do you think? |  |
| Too few questions | 20 (4.1) |
| About the right number of questions | 413 (85.2) |
| Too many questions | 52 (10.7) |
| Did you find yourself focusing on one or more of the characteristics when making your choices? *(multiple choices possible)* |  |
| The health professional Sam will see | 265 (54.6) |
| Type of consultation | 244 (50.3) |
| What information will be provided | 181 (37.3) |
| Incentives to engage with preconception care | 115 (23.7) |
| The out-of-pocket costs to Sam | 288 (59.4) |
| I considered none of the characteristics | 11 (2.3) |

# DIRECT checklist

Table S8. Checklist for reporting discrete choice experiments in health

| **Section item** | | **Page and Paragraph** |
| --- | --- | --- |
| **Purpose and Rationale** | |  |
| 1 | Describe the real-world context and decision-maker that the hypothetical choice context seeks to replicate or inform | Manuscript: Introduction |
| 2 | Provide a rationale for using a DCE to answer the research question | Manuscript: Introduction |
| **Attributes and levels** | |  |
| 3 | Describe how attributes and levels were derived (e.g. literature review, interviews, focus groups, expert input) | Manuscript: Section 2.2 - Identification of attributes and levels |
| 4 | Provide the final list of attributes and levels | Manuscript: Table 1 |
| **Experimental design** | |  |
| 5 | Report the number of alternatives per choice set and whether they were labelled or unlabelled | Manuscript: Section 2.2 - Identification of attributes and levels |
| 6 | Describe response options (e.g. forced choice, opt-out, status quo) | Manuscript: Section 2.4 – Data collection |
| 7 | Describe the type of experimental design (e.g. orthogonal, D-efficient, Bayesian efficient, partial profile) | Manuscript: Section 2.2 - Identification of attributes and levels; Supplementary material: Section 1– Details about the designed experiment |
| 8 | Describe which effects are identified in the design (e.g. main effects, higher order interactions, functional form) | Supplementary material: Section 1 – Details about the designed experiment |
| 9 | Describe the number of choice sets, blocks and choice sets per block | Manuscript: Section 2.2 - Identification of attributes and levels; Supplementary material: Section 1– Details about the designed experiment |
| 10 | Indicate how the experimental design was obtained (software, catalogue, other) | Supplementary material: Section 1 – Details about the designed experiment |
| **Survey design** | |  |
| 11 | Provide a sample choice set and the instructions and background information given to respondents (e.g. providing the survey as an appendix) | Manuscript: Section 2.3 - Data Collection, Figure 1; Supplementary material: Section 9 – Example of full survey |
| 12 | Report any randomisation (e.g. choice set order, attribute order, alternative order, framing effects) | Manuscript: Section 2.2 - Identification of attributes and levels; Supplementary material: Section 1– Details about the designed experiment |
| 13 | Describe what was checked in piloting (e.g. understanding, respondent burden, timing, wording) | Manuscript: Section 2.3 - Data Collection |
| 14 | Report whether information from the pilot was used to update the experimental design (e.g. priors, functional form of attributes) or survey design functional form of attributes) or survey design | Manuscript: Section 2.3 - Data Collection |
| **Sample and data collection** | |  |
| 15 | Report respondent inclusion/exclusion criteria | Manuscript: Section 2.4 – Study sample |
| 16 | Describe how data were collected (e.g. mail, personal interview, web survey) | Manuscript: Section 2.4 – Study sample |
| 17 | Report the response rate or cooperation rate, if possible | Not provided by survey provider |
| 18 | Report the final sample size and how the sample size was determined | Manuscript: Section 2.4 – Study sample |
| 19 | Describe respondent characteristics and representativeness of target population, if known | Manuscript: Section 3.1 – Characteristics of the respondents; Supplementary material: Section 2 |
| **Econometric analysis** | |  |
| 20 | Indicate coding of data (e.g. effects, dummy, continuous) including definitions | Manuscript: Section 2.5 - Statistical analysis |
| 21 | Report whether any respondents were removed and why (e.g. suspected fraudulent responses, rationality tests) | Manuscript: Section 3.3 – Analysis of choice tasks |
| 22 | Provide the rationale for model choice (e.g. conditional logit, mixed logit, latent class) and assumptions (e.g. error variance) | Manuscript: Section 2.5 - Statistical analysis |
| 23 | Report model specification | Manuscript: Section 2.5 – Statistical Analysis; Supplementary material: Section 4, Section 5, Section 6 |
| **Reporting of results** | |  |
| 24 | Report the model performance, goodness of fit (if comparing models) | Supplementary material: Section 4, Section 5, Section 6 |
| 25 | Describe methods used for analysis of model results (e.g. calculation of marginal rate of substitution, attribute relative importance, welfare gain) | Manuscript: Section 2.5 – Statistical Analysis, Section 3 - Results |
| 26 | Report measures of precision for the output(s) of interest (e.g. confidence intervals) and how these were derived | Manuscript: Section 2.5 – Statistical Analysis; Supplementary material: Section 4, Section 5, Section 6 |
| Source: Ride J, Goranitis I, Meng Y, LaBond C, Lancsar E. A Reporting Checklist for Discrete Choice Experiments in Health: The DIRECT Checklist. *Pharmacoeconomics*. 2024;42(10):1161-1175. doi:10.1007/s40273-024-01431-6 | | |

# **Example of a version of the full survey**

**Survey:** Preconception care Discrete Choice Experiment

**Survey provider:** [Pureprofile](https://www.pureprofile.com/)

**Date of collection:** August 2023

**Conducted by:** Centre for Health Economics Research and Evaluation, Faculty of Health,
University of Technology Sydney


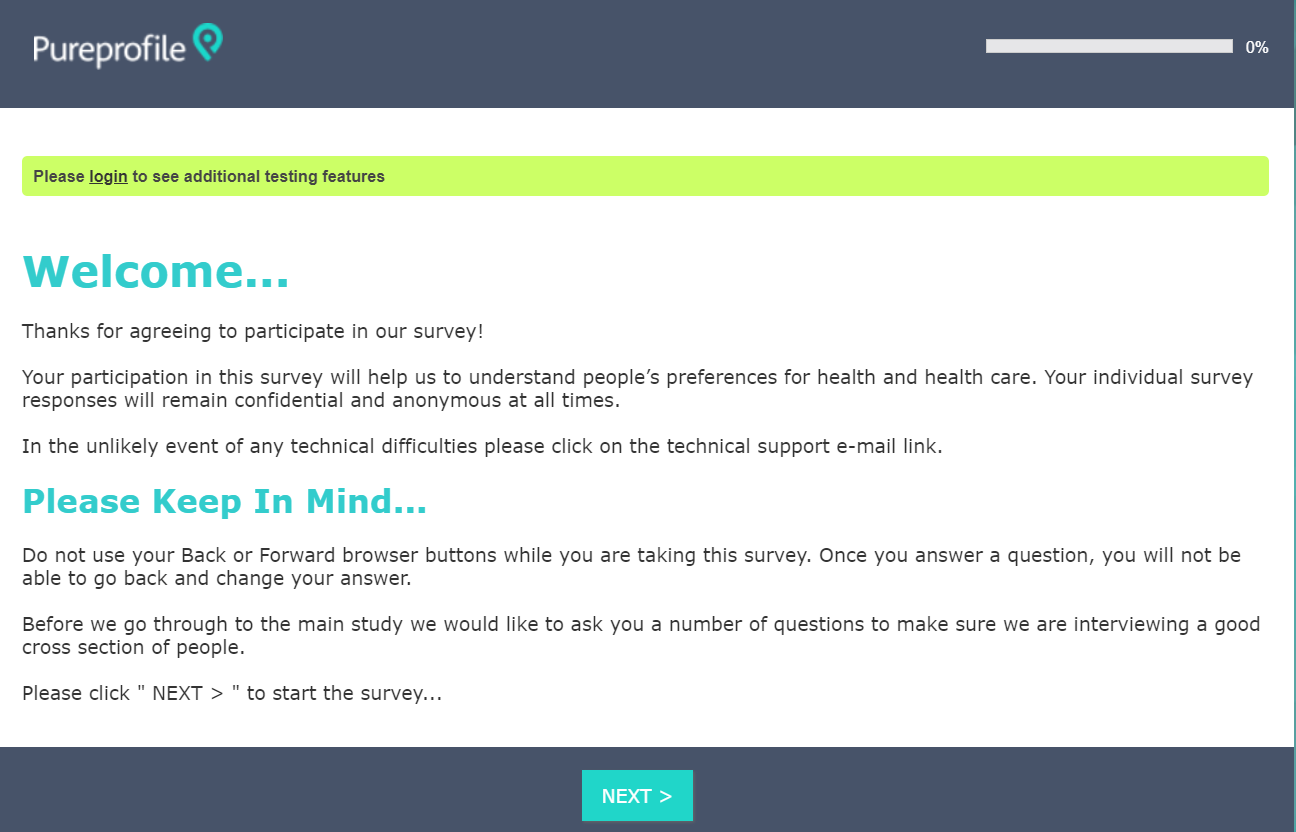


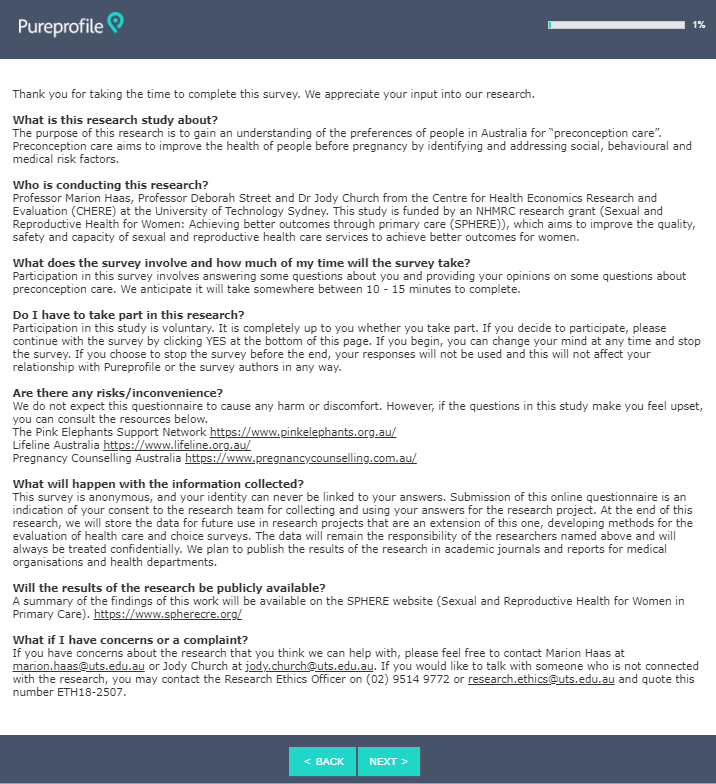


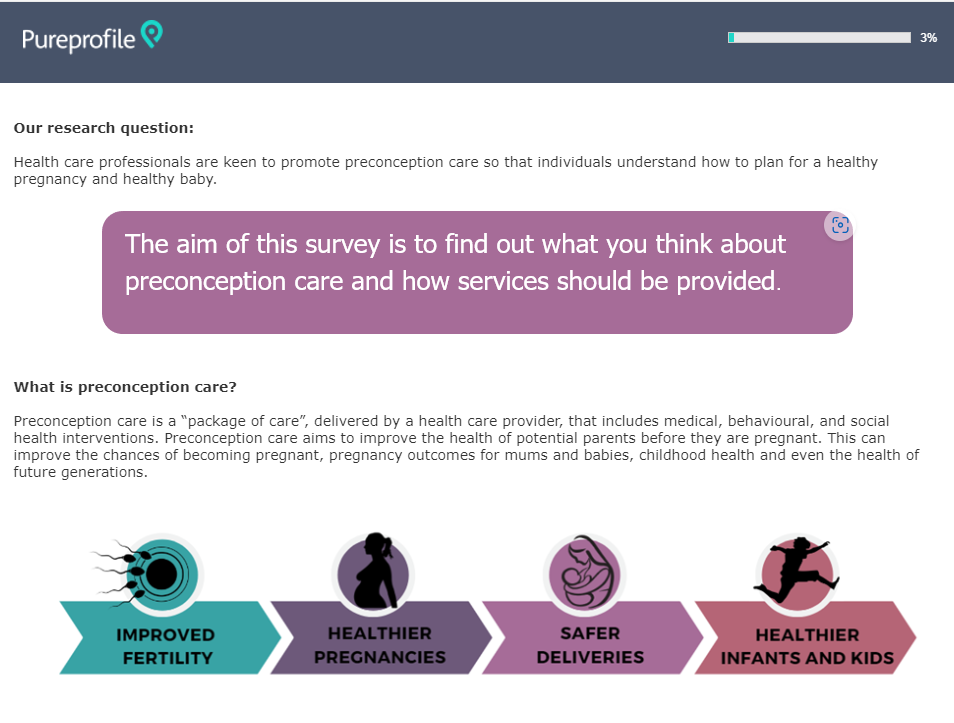


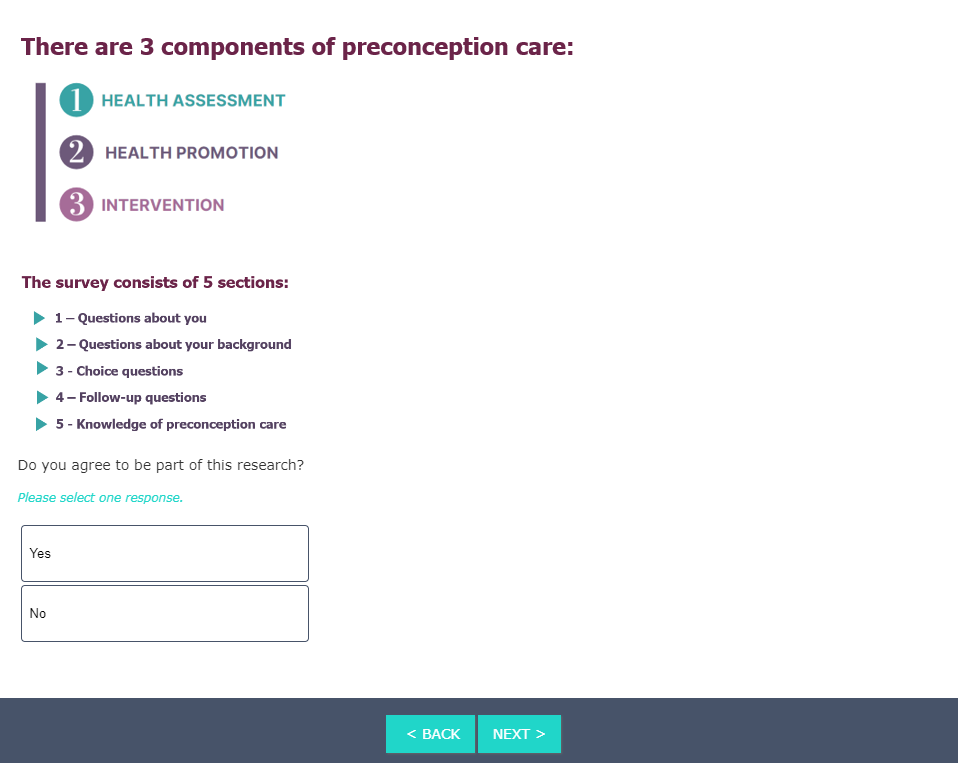


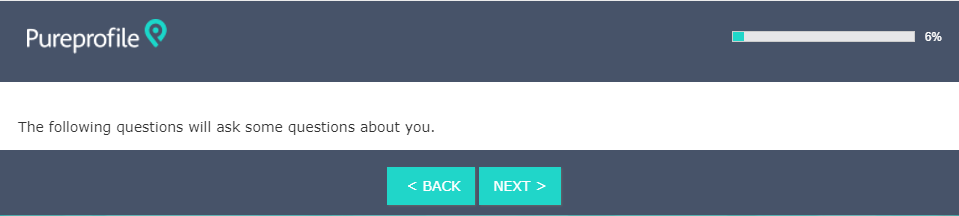


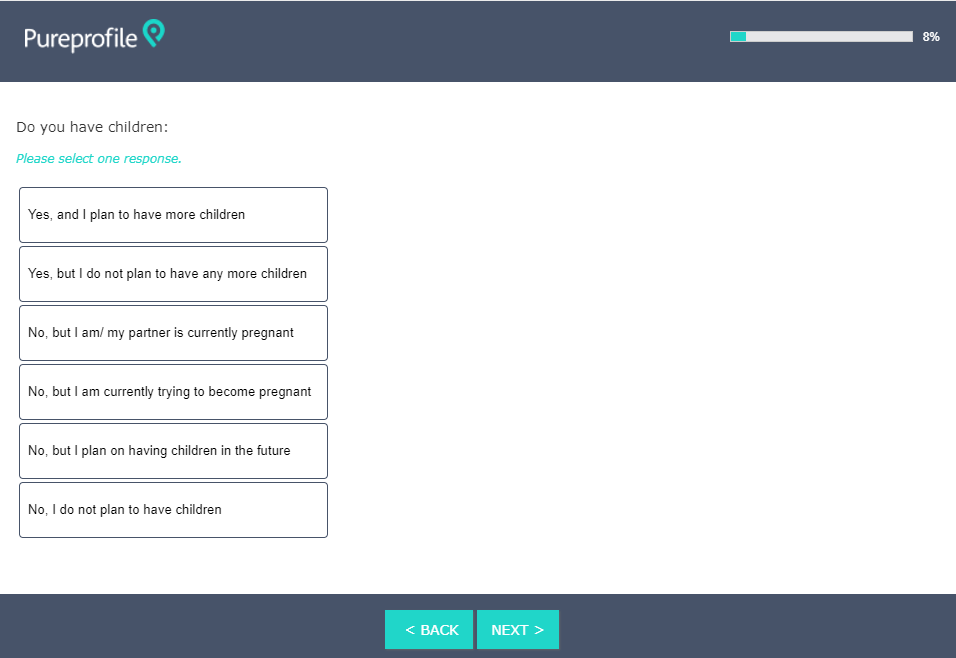


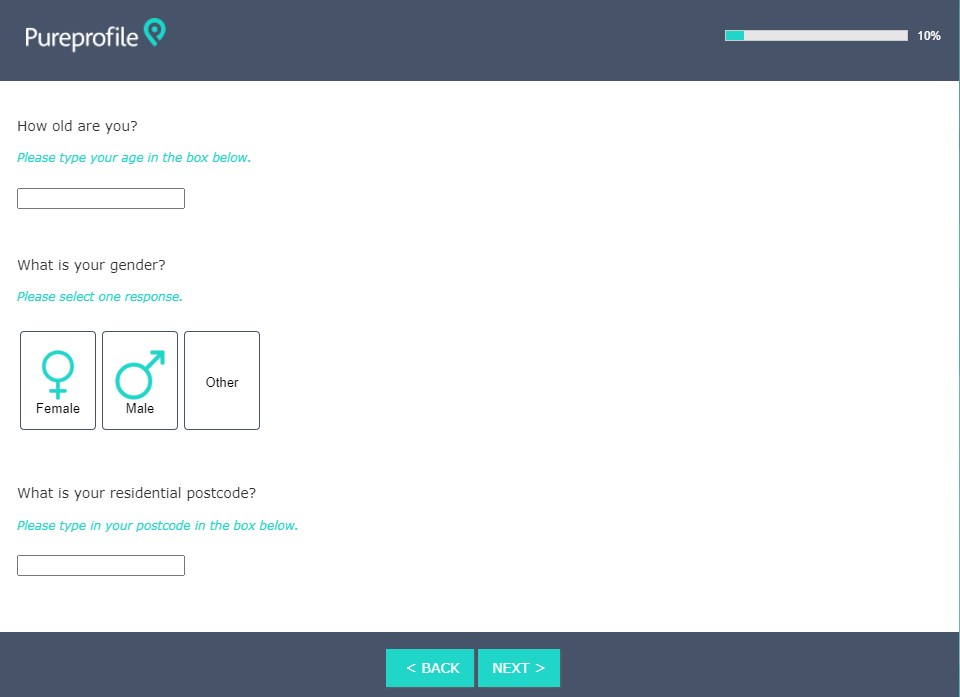


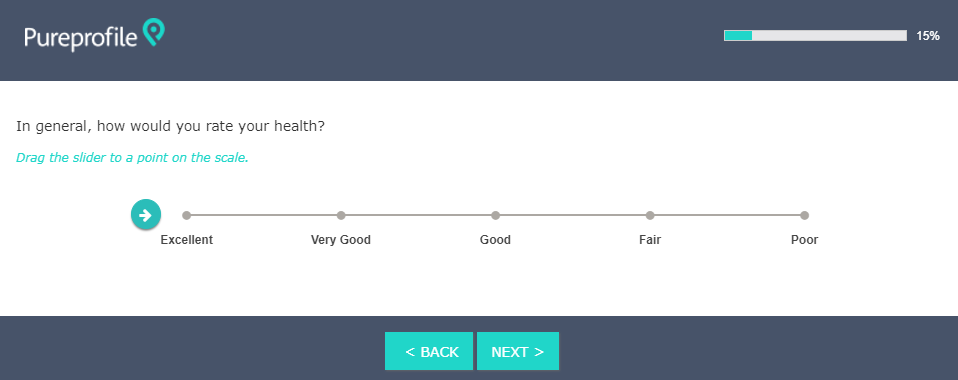


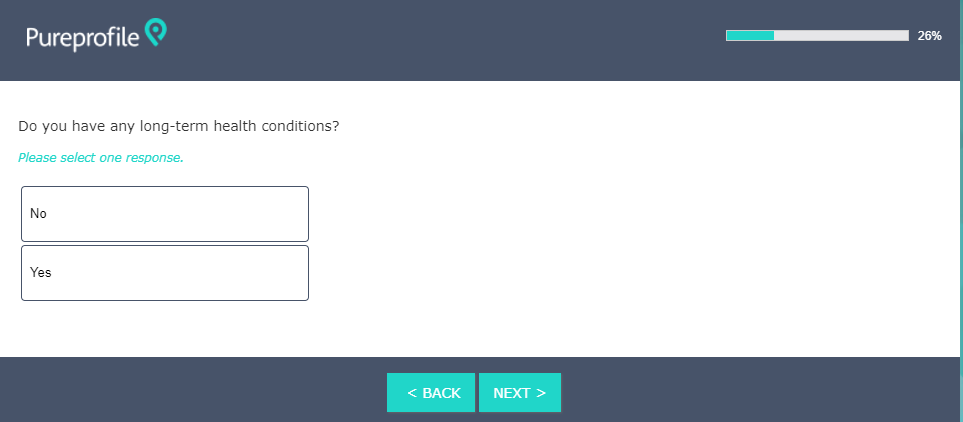


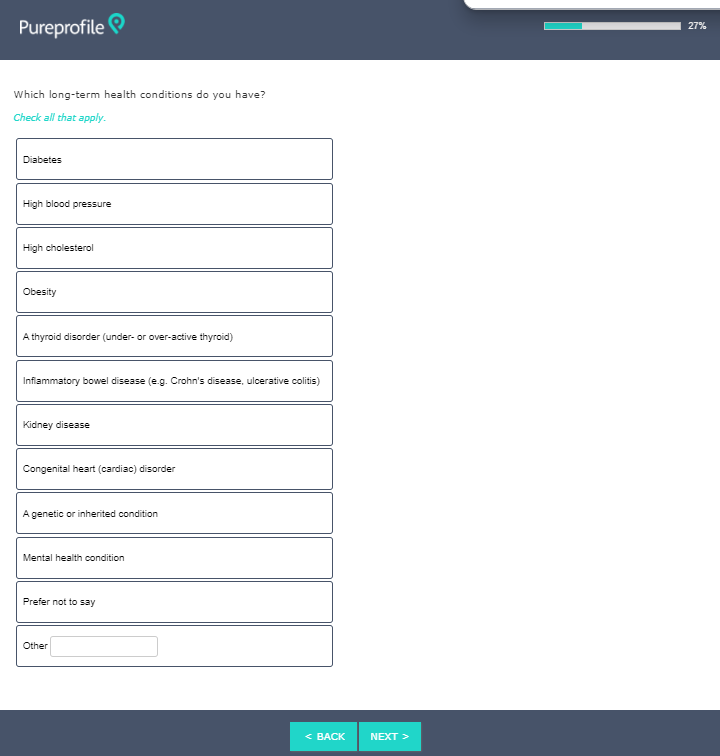


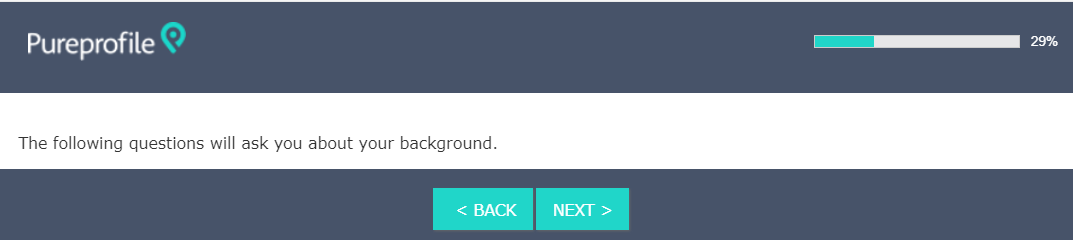


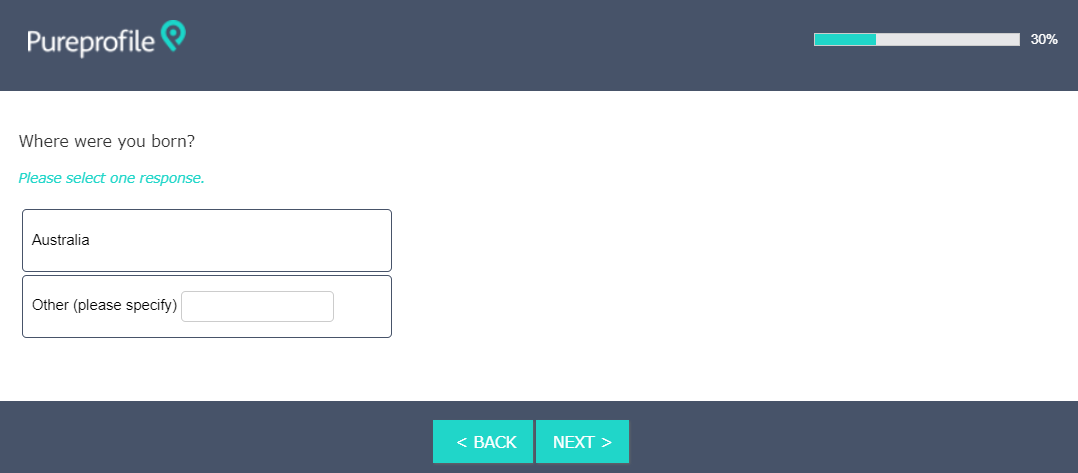


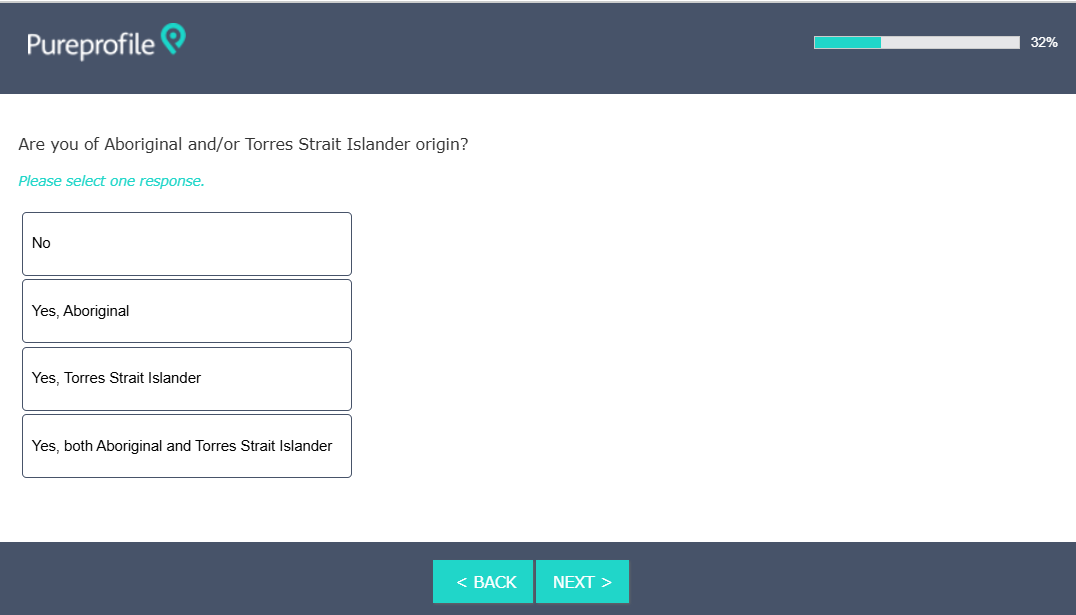


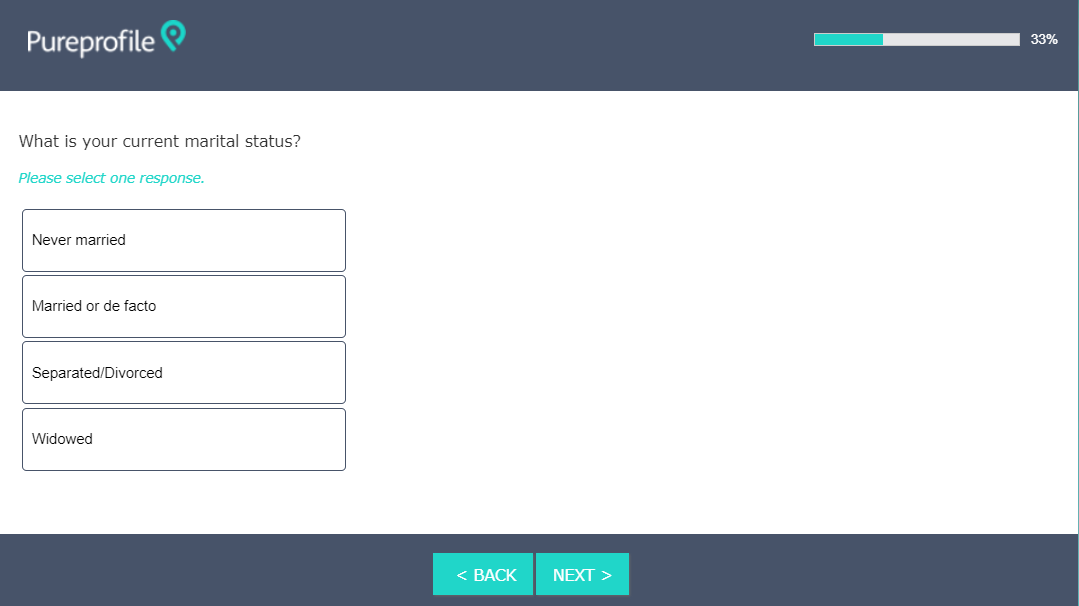


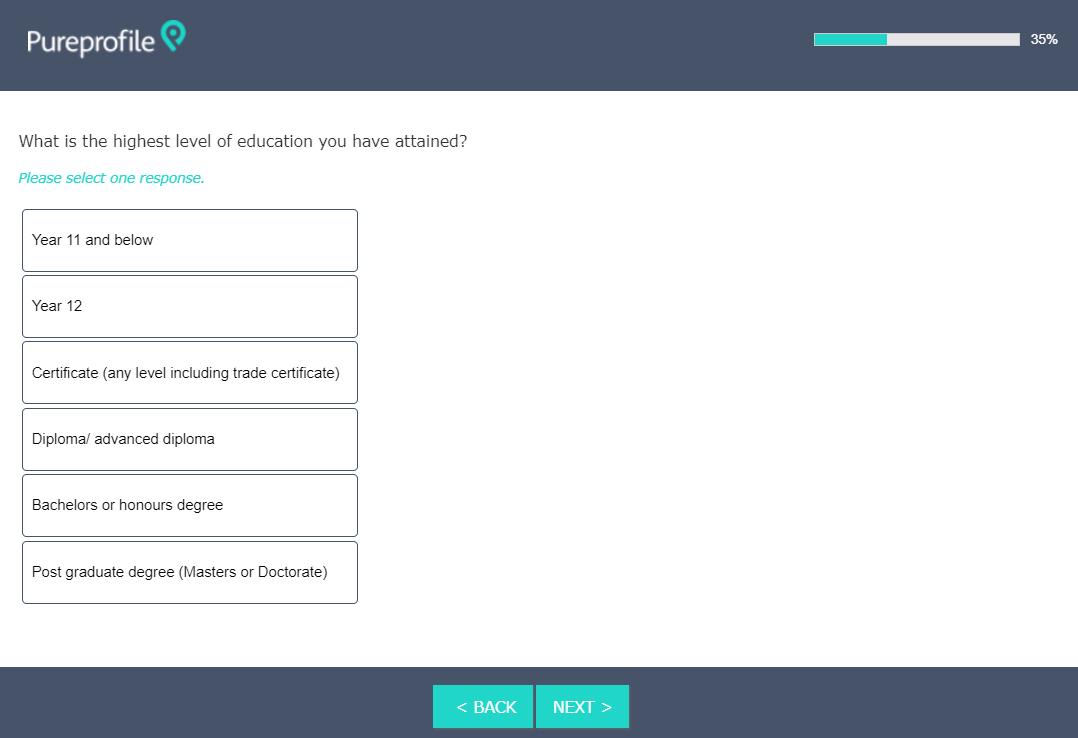


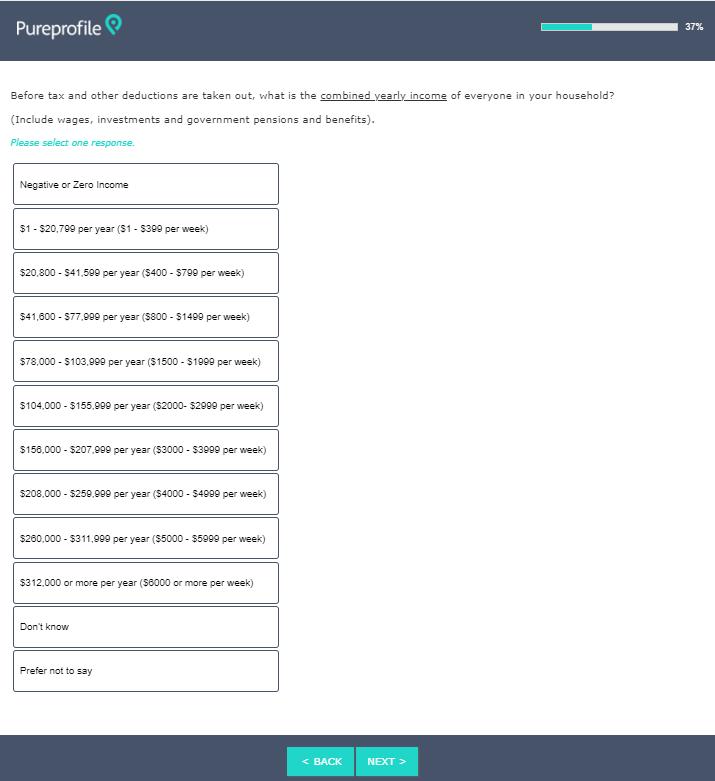


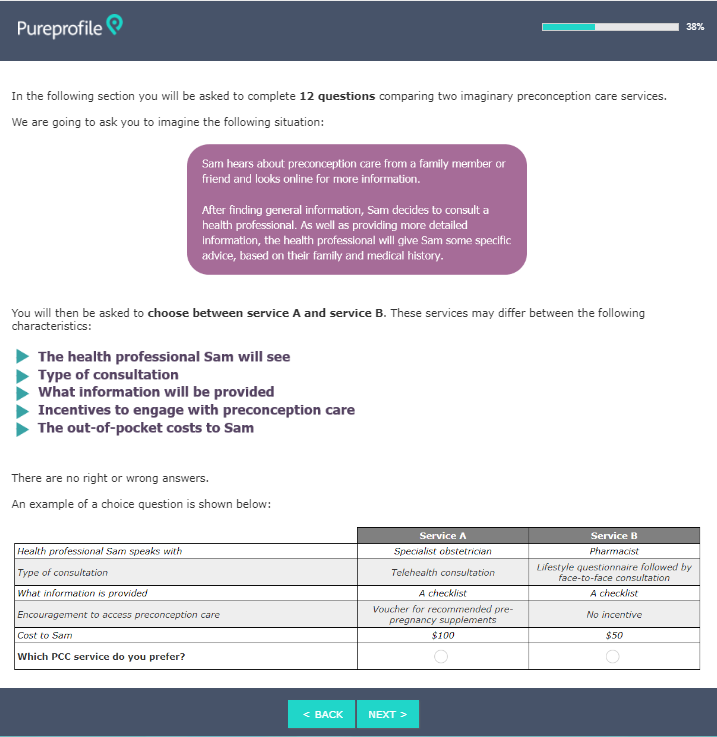


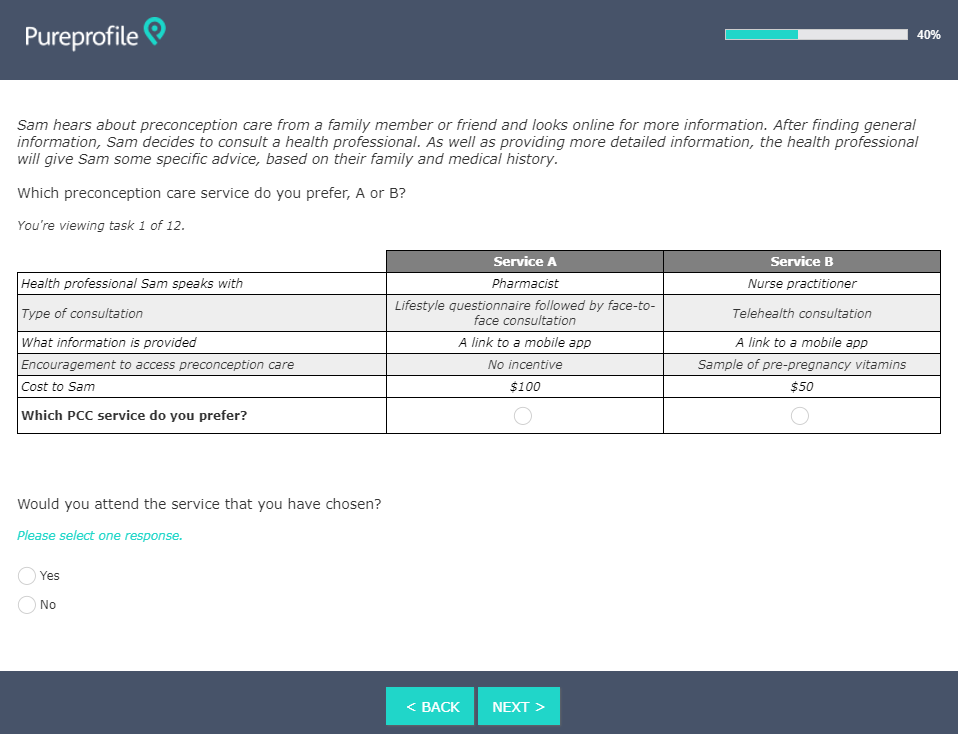


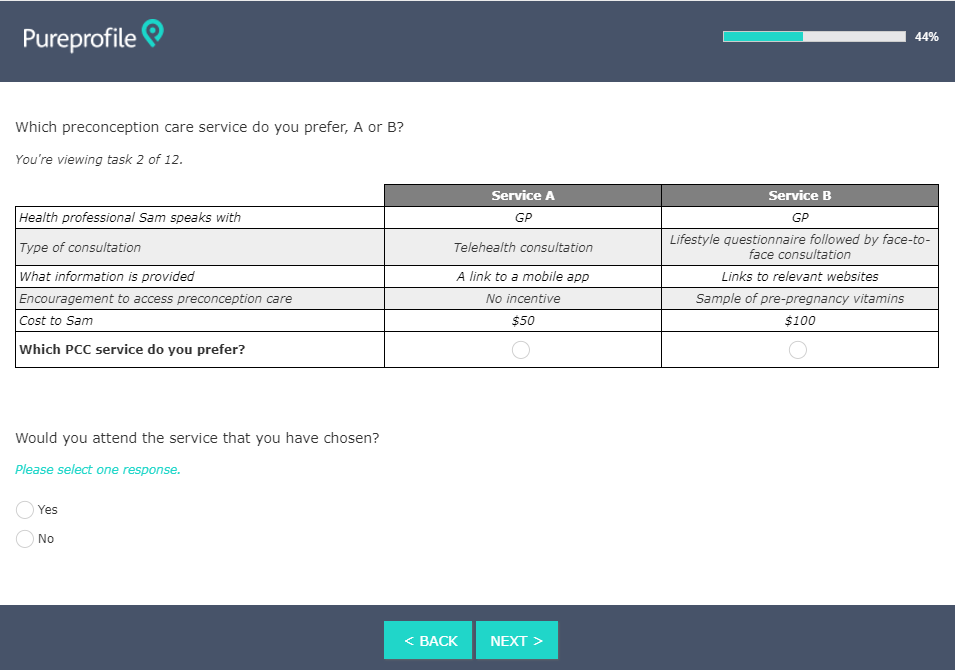


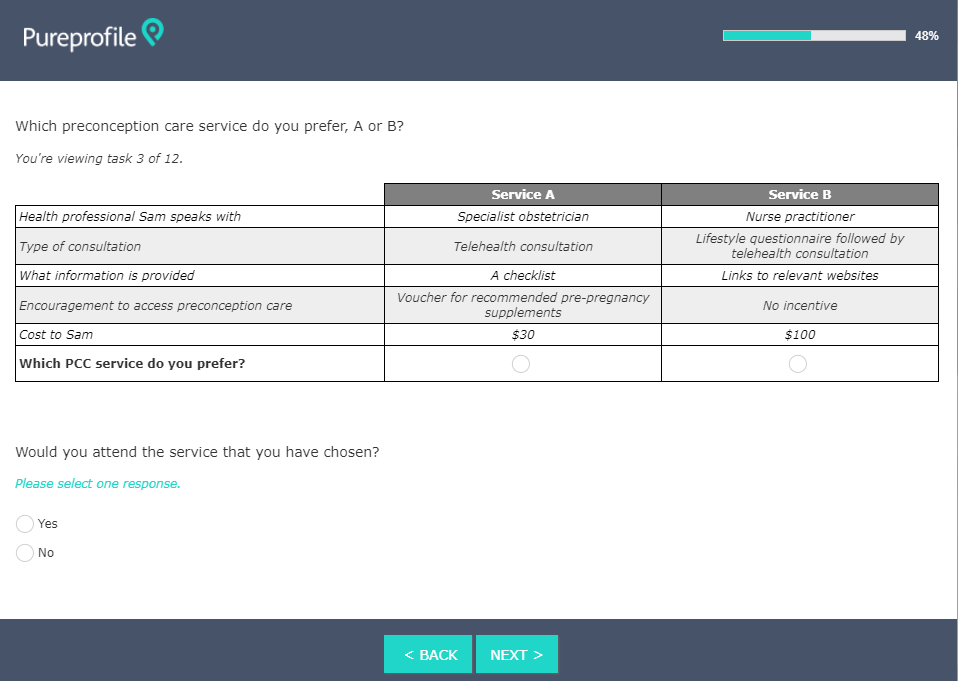


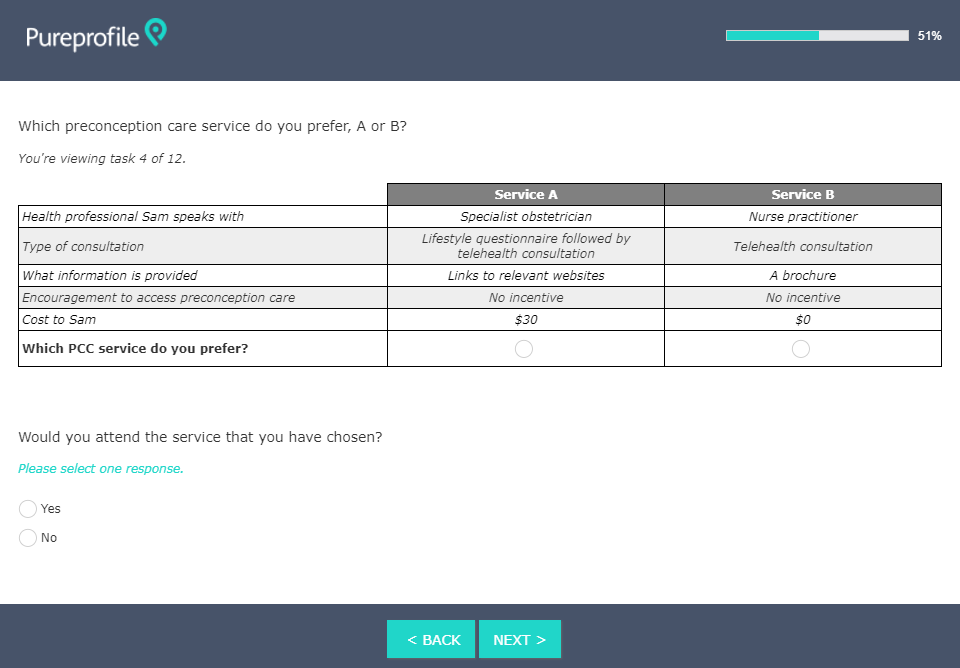


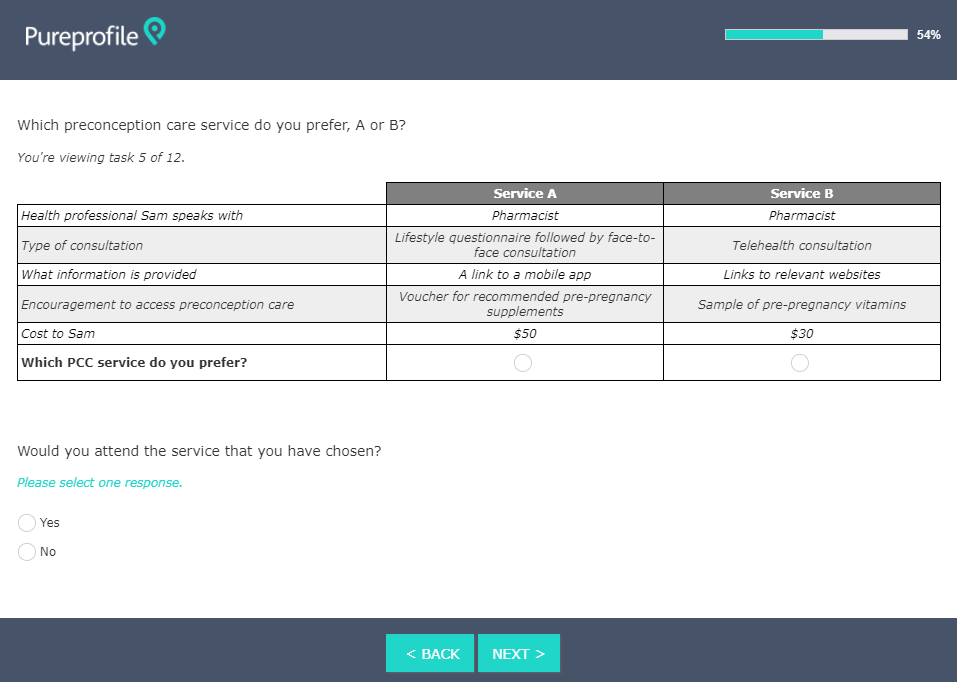


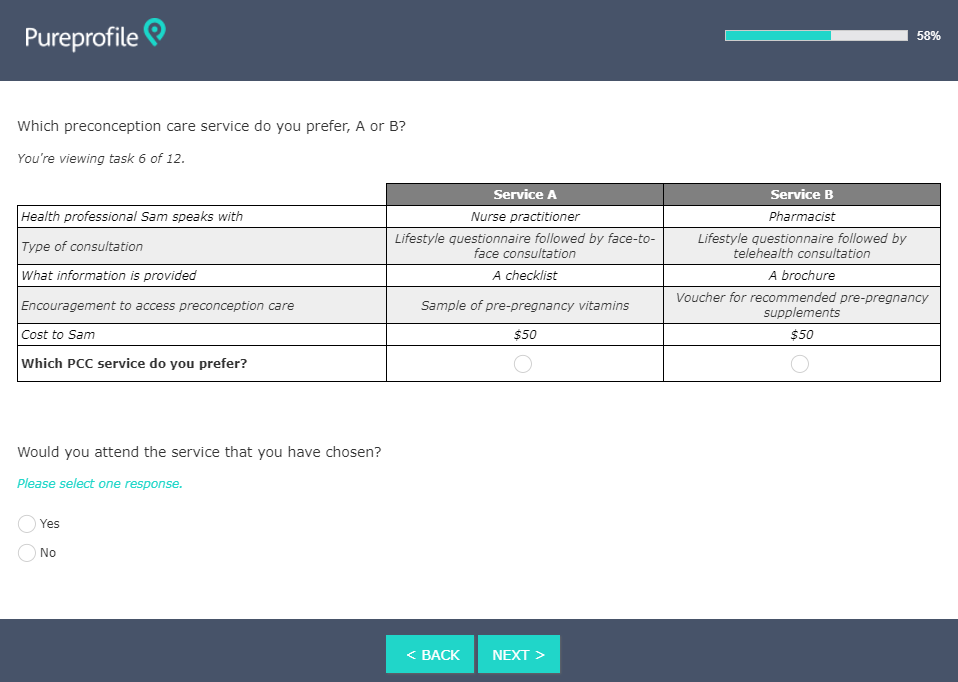


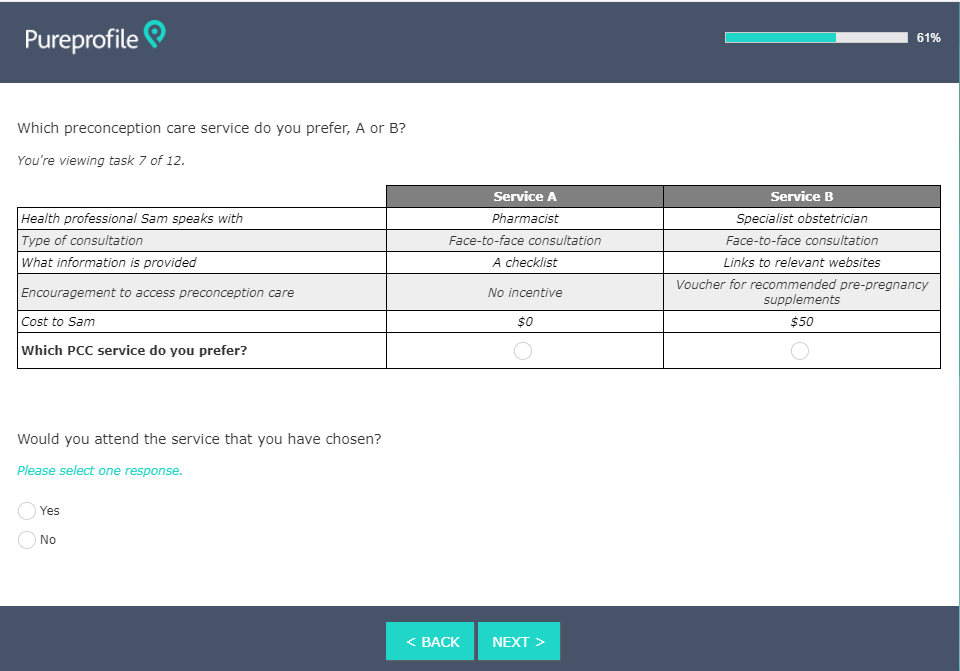


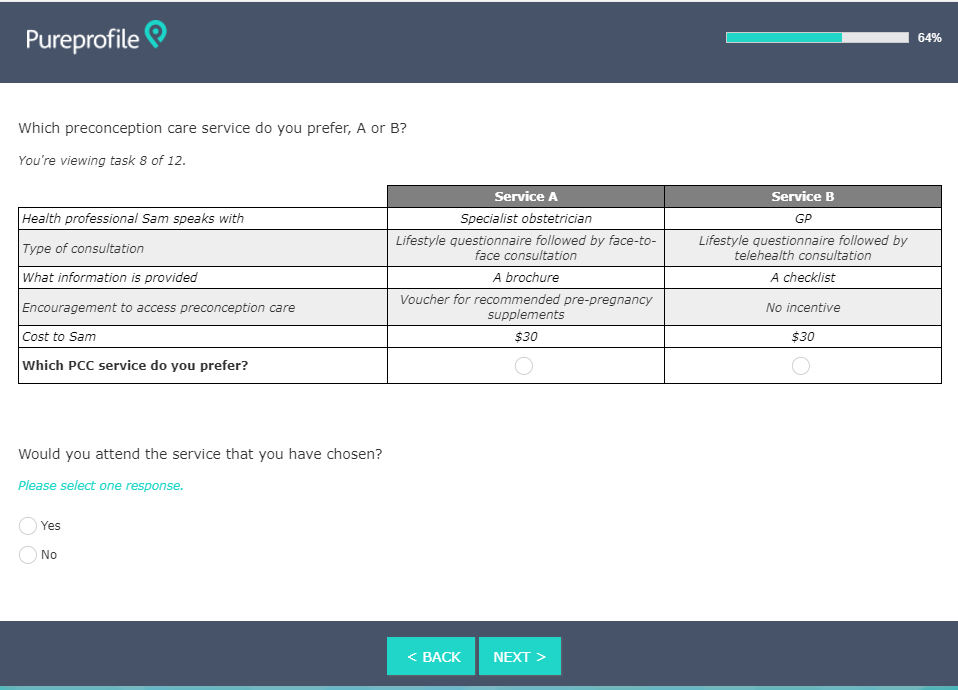


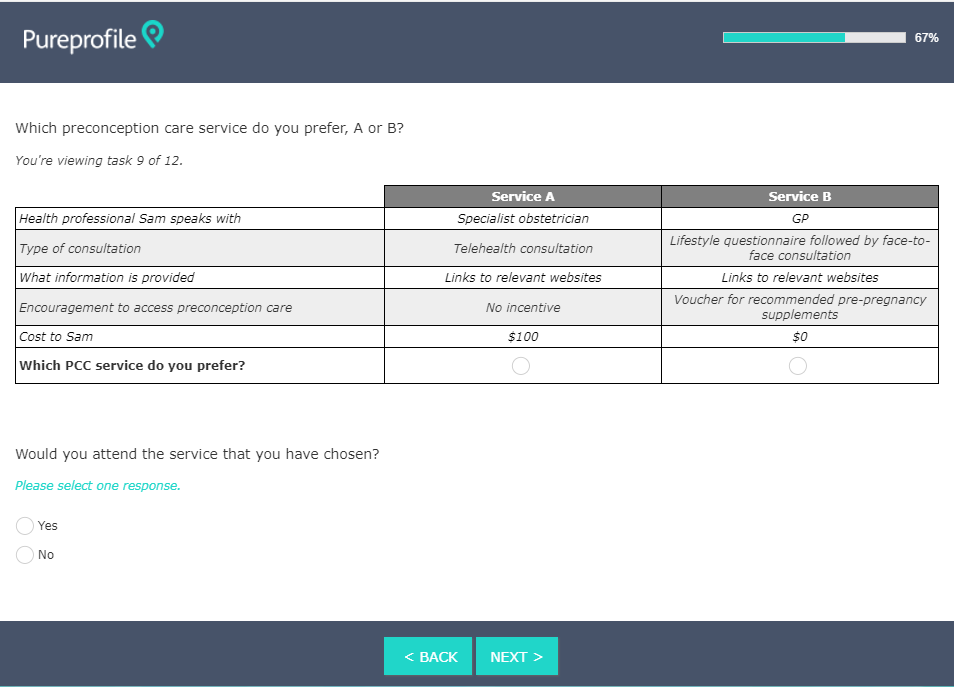


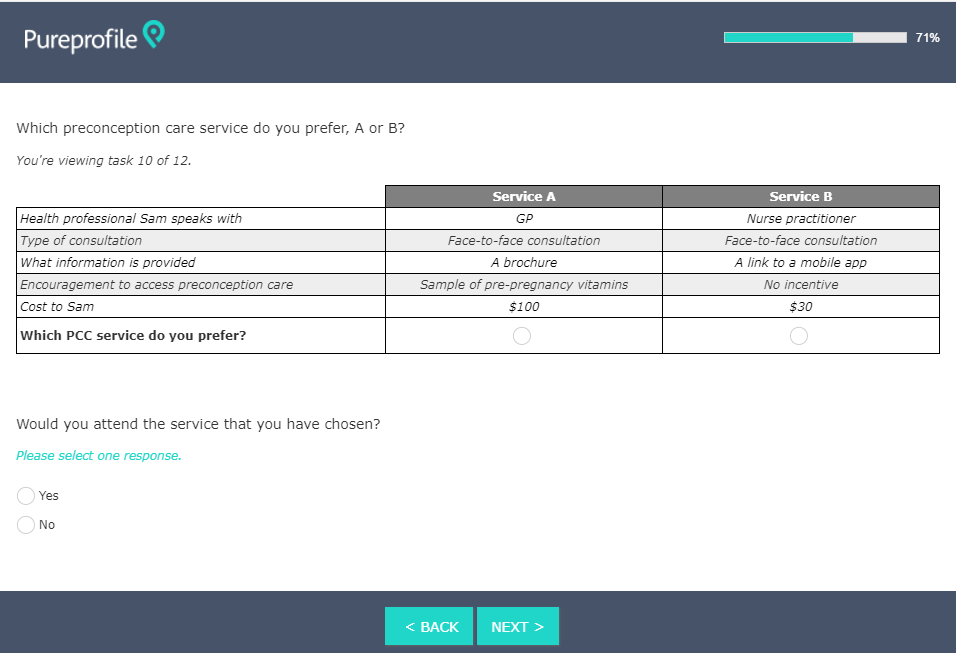


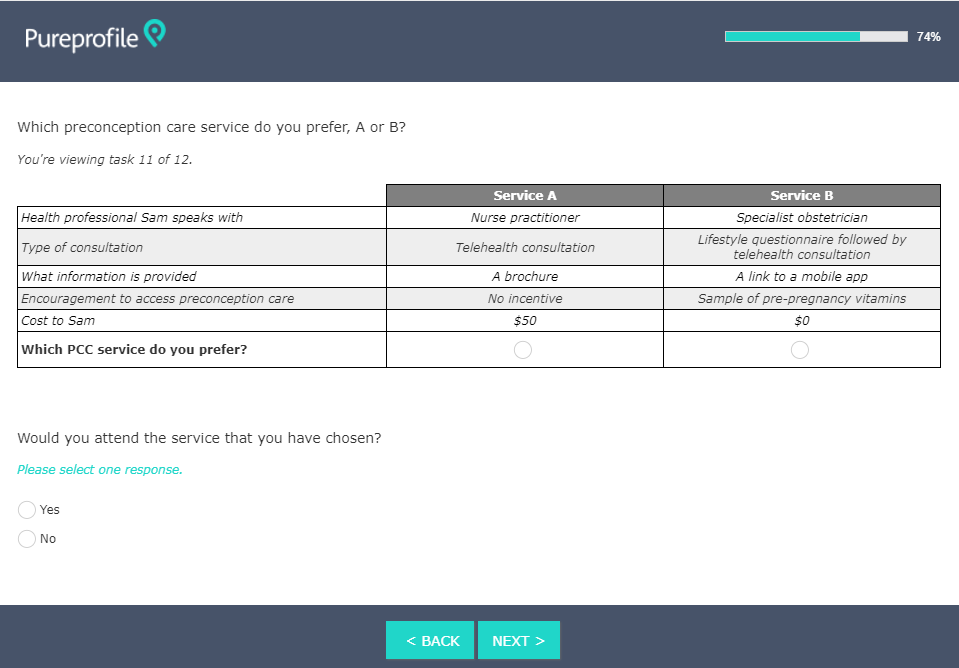


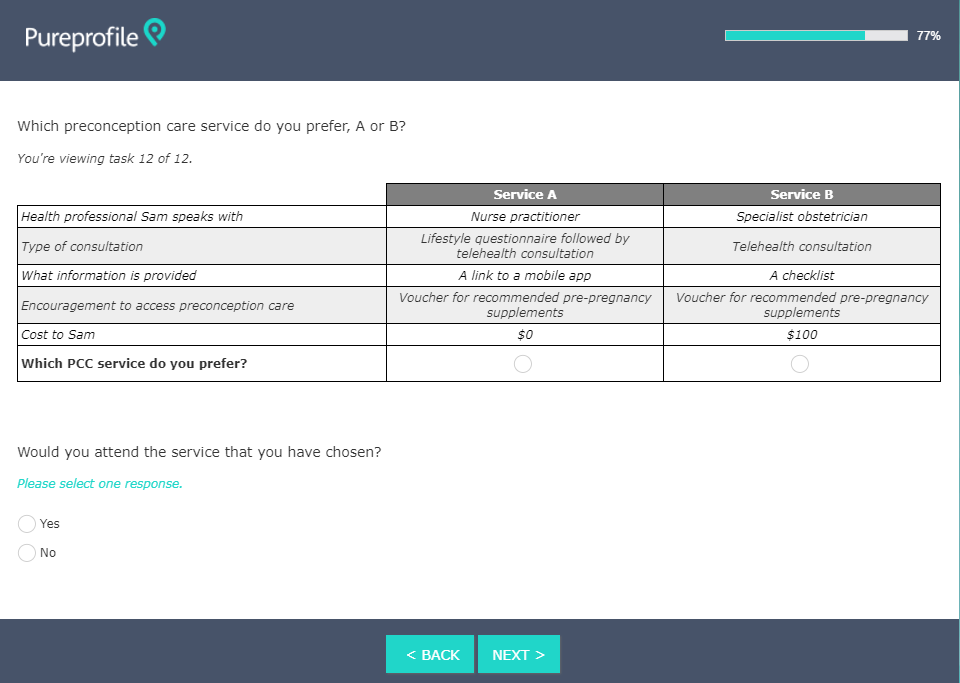


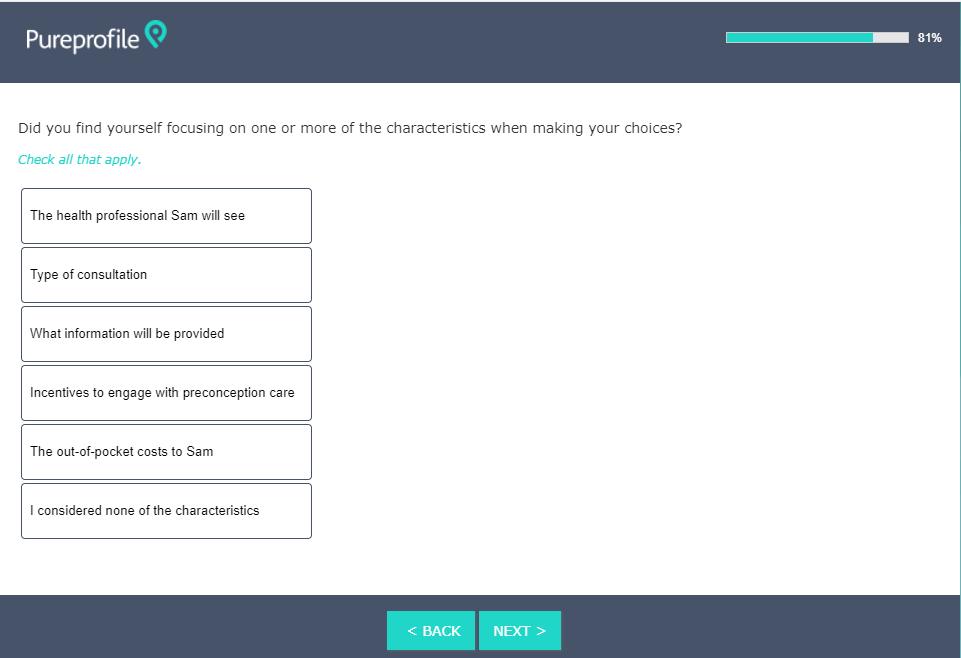


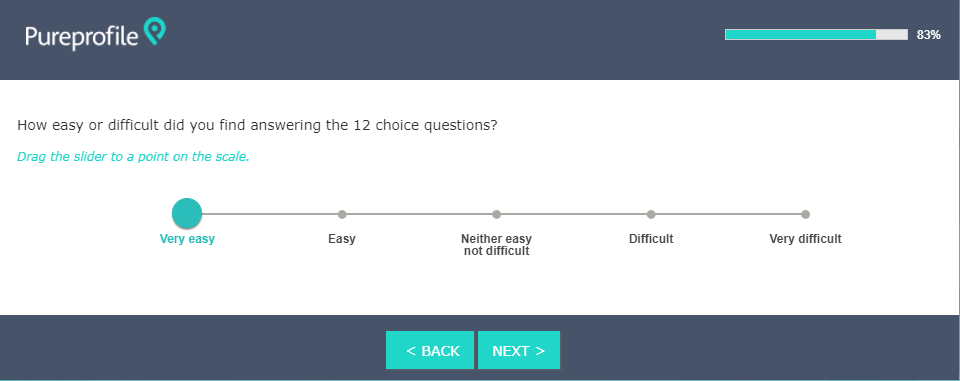


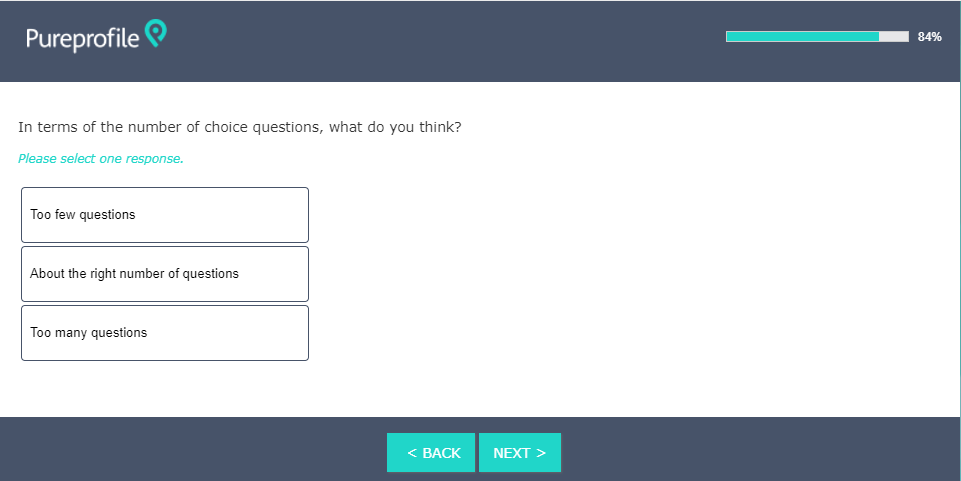


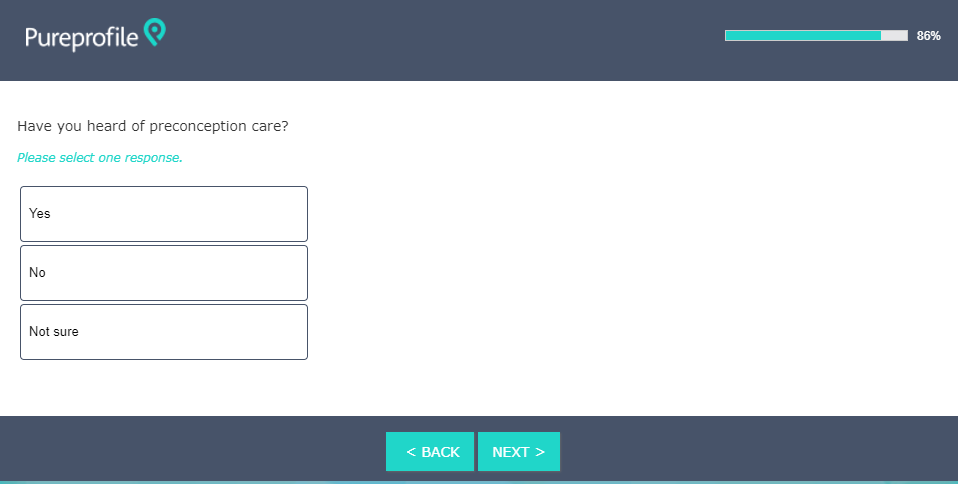


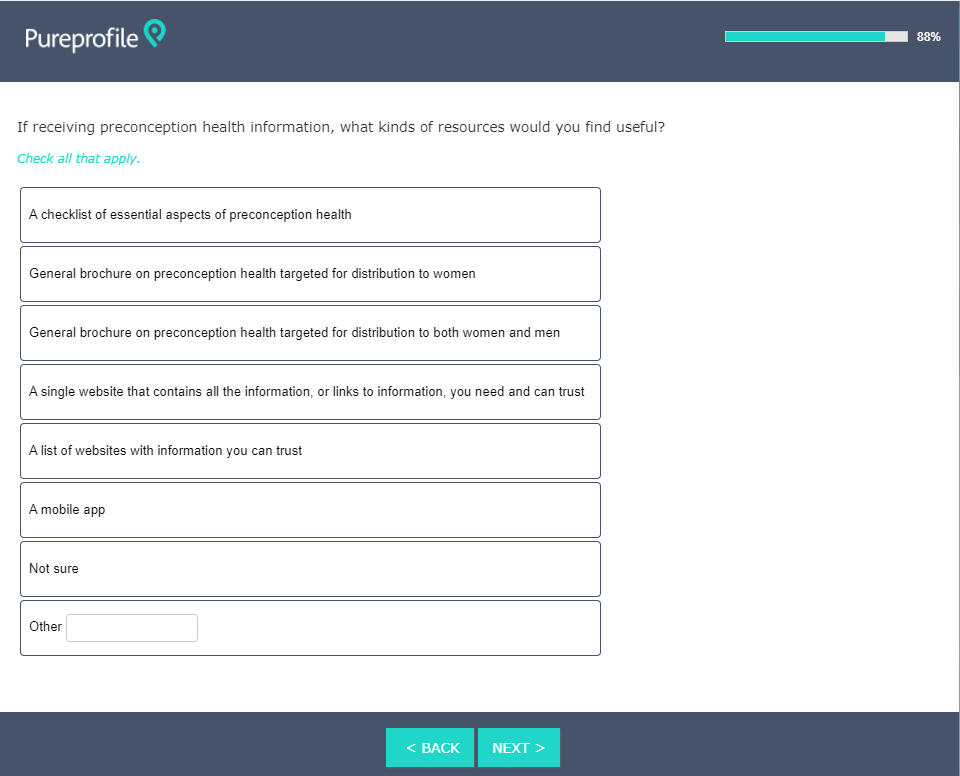


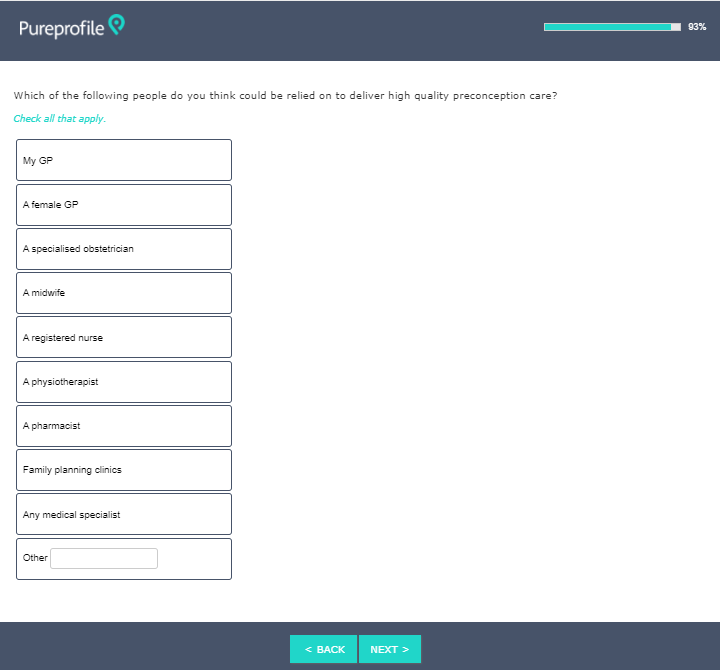


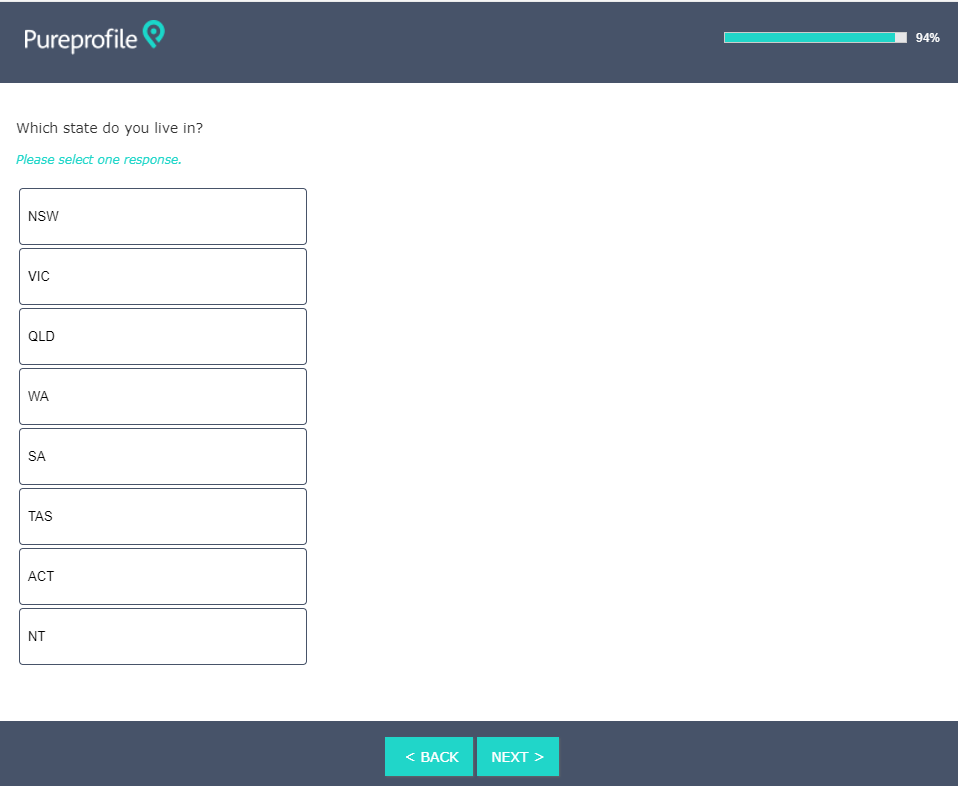


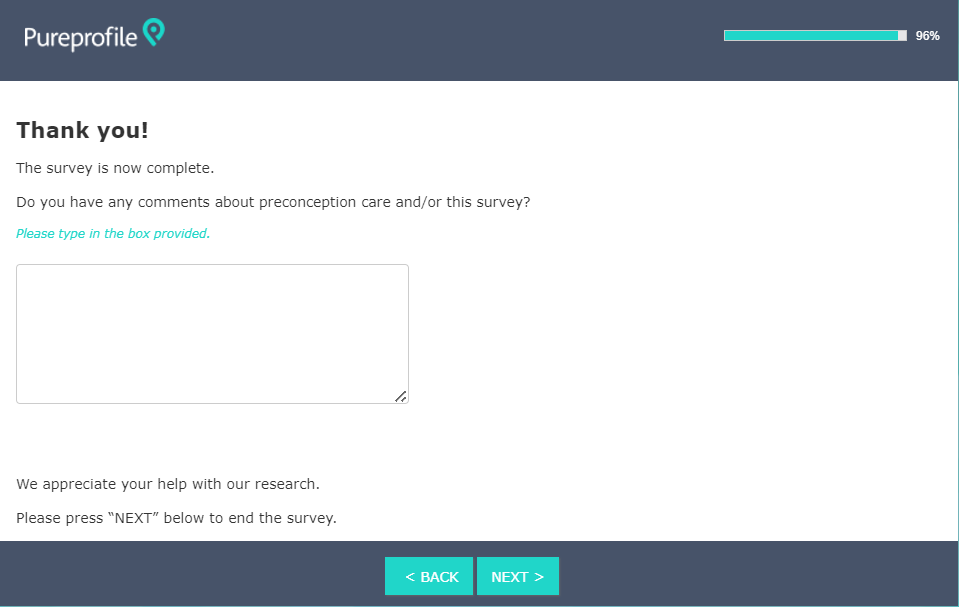


1. Kuhfeld, WF (2006) Orthogonal arrays. Technical report, SAS Institute. Available at <https://support.sas.com/techsup/technote/ts723_Designs.txt> [↑](#footnote-ref-2)
